# Characterizing heterogeneity in early adolescent reward networks and individualized associations with behavioral and clinical outcomes

**DOI:** 10.1162/netn_a_00306

**Published:** 2023-06-30

**Authors:** Matthew Mattoni, David V. Smith, Thomas M. Olino

**Affiliations:** Department of Psychology and Neuroscience, Temple University, Philadelphia, PA, USA

**Keywords:** Effective connectivity, Heterogeneity, Adolescence, Reward, Depression, Substance use

## Abstract

Associations between connectivity networks and behavioral outcomes such as depression are typically examined by comparing average networks between known groups. However, neural heterogeneity within groups may limit the ability to make inferences at the individual level as qualitatively distinct processes across individuals may be obscured in group averages. This study characterizes the heterogeneity of effective connectivity reward networks among 103 early adolescents and examines associations between individualized features and multiple behavioral and clinical outcomes. To characterize network heterogeneity, we used extended unified structural equation modeling to identify effective connectivity networks for each individual and an aggregate network. We found that an aggregate reward network was a poor representation of individuals, with most individual-level networks sharing less than 50% of the group-level network paths. We then used Group Iterative Multiple Model Estimation to identify a group-level network, subgroups of individuals with similar networks, and individual-level networks. We identified three subgroups that appear to reflect differences in network maturity, but this solution had modest validity. Finally, we found numerous associations between individual-specific connectivity features and behavioral reward functioning and risk for substance use disorders. We suggest that accounting for heterogeneity is necessary to use connectivity networks for inferences precise to the individual.

## Author Summary

Associations between brain connectivity and behavioral phenotypes are typically examined by comparing group averages (e.g., clinical vs. control). This approach presumes that the group-average network reflects individuals. However, if individuals’ network structures are highly heterogenous, averaging across them will create a group-level network that does not generalize to individuals, preventing valid inferences of associations with behavioral differences. Here, we showed that a group-level reward network in early adolescents poorly reflected individuals. We then used GIMME to identify reward network features that were specific to individuals. These network features were associated with multiple reward-related outcomes, including familial risk for substance use disorder (*R*^2^ = 31%). A focus on the individual, rather than groups, may be necessary for valid inferences of individual behavioral differences from fMRI connectivity.

## INTRODUCTION

Adolescent reward network processing is associated with several health-related outcomes, including risk-taking behaviors, depression, and substance use problems (Casey et al., 2008). Research has traditionally compared aggregate network models between known groups, such as individuals with and without target disorders. This approach implicitly assumes that each group is a homogenous population such that individuals can be represented by a single aggregate network. However, there is increasing evidence that there is substantial heterogeneity in neural networks within groups that are defined by a single behavioral phenotype (Drysdale et al., 2017; Feczko & Fair, 2020; Marquand et al., 2016; Price et al., 2017b). This heterogeneity may result in a group-average model that is not representative of some, or all, individuals, limiting the ability to use a case-controlled framework to make inferences at the individual level. Alternatively, estimating networks that are more precise to the individual can improve the ability to make inferences specific to the individual, rather than the group. In this study, we parse heterogeneity in adolescent effective connectivity reward networks using multiple methods and examine associations between individual-specific network features and reward-related behavioral outcomes. First, we examine the applicability of the aggregate network structure to that for each individual. Second, we derive subgroups of participants who share similar connectivity network features. Finally, we examine how features of networks, including subgroups and individual connectivity paths, are associated with outcomes including reward-related behaviors, depression, and risk for alcohol and substance use problems.

Case-controlled study designs can provide insight into broad neural differences between groups. However, translational utility of neuroimaging (e.g., diagnosis, treatment selection, etc.) ultimately requires that models exhibit group-to-individual generalizability. Model group-to-individual generalizability is termed ergodicity, and requires that individuals conform to a similar model (Fisher et al., 2018; Molenaar, 2004). Early examinations of neural networks have found that individuals exhibit qualitatively distinct traitlike patterns that are not reflected by an aggregate neural model (Gratton et al., 2020; Laumann et al., 2015; Medaglia et al., 2011; Seitzman et al., 2019), preventing group-to-individual generalizability. The inability to make inferences on an individual level may be a key factor in the limited clinical utility of fMRI scans (Zhuo et al., 2019), despite the numerous reviews and meta-analyses that have implicated neural reward processing dysfunction in adolescent depression at a group level (Fischer et al., 2019; Keren et al., 2018; Miller et al., 2015; O’Callaghan & Stringaris, 2019). Furthermore, heterogeneity across individuals may also limit inferences in nonclinical cognitive neuroscience research if the group-level model obscures qualitative differences in network functioning which may represent distinct psychological processes across individuals (e.g., Demidenko et al., 2022).

One alternative approach to case-controlled designs is identifying biological subgroups that represent more homogenous network patterns across individuals and then examining behavioral differences between them (Feczko & Fair, 2020). Thus, subgrouping approaches identify groups of individuals based on similarity in network functioning, rather than behavior (e.g., depression). Furthermore, as subgroups serve as an intermediate between the individual and the group, they may be more representative of specific individuals than group-level models, and increase the precision of resulting inferences to the individual. Multiple studies have used subgroups to identify more precise neural network associations with depression (Drysdale et al., 2017; Liang et al., 2020), attention deficit/hyperactivity disorder (Costa Dias et al., 2015), and alcohol and substance use disorders (Kashyap et al., 2020; Zhu et al., 2022). These studies (further reviewed in the Supporting Information) have demonstrated that subgroups have external validity in their associations with clinical functioning. Moreover, as they are intended to represent individuals more similar in network models, they may better represent individuals than group-level models. However, few studies have assessed the internal validity of the identified subgroup solution (Brucar et al., 2023), an important step to ensure that the identified subgroups are capturing true network differences, rather than noise.

The development of Group Iterative Multiple Model Estimation (GIMME; Beltz & Gates, 2017; Gates et al., 2014; Gates & Molenaar, 2012) has furthered research examining network heterogeneity by identifying group-level networks with paths common to the sample, data-driven subgroups, and individualized networks. GIMME searches for unobserved network structures in intensive time series data. Rather than averaging across participants, GIMME only adds paths to a group-level network that are statistically significant for the majority of individuals in a specified sample. GIMME then uses a community detection algorithm to also search for subgroups of individuals with similar network features, and, finally, adds paths that are significant for each individual considered independently.

Several studies have used GIMME to study associations between subgroups of network functioning and clinical outcomes. Price et al. (2017a) used GIMME to identify two subgroups of resting-state connectivity in a sample of adults with depression. They found that a subgroup with an intradefault mode network (DMN) path from the perigenual anterior cingulate cortex to the posterior cingulate cortex (PCC) and a path from the dorsal anterior cingulate cortex (ACC) to the right insula represented the majority of patients, and a subgroup defined by additional paths to the parietal lobe represented more participants who identified as female, had comorbid anxiety disorders, and had more recurrent depression. In a separate study of adults with and without depression, Price et al. (2017b) identified two subgroups of network connectivity after positive mood induction. They found that one subgroup had fewer connectivity paths and decreased connectivity in ventral affective network and DMN paths relative to the other subgroup, and individuals in this subgroup had a higher rate of depression diagnosis and higher symptom severity. While most studies have focused on adult samples and resting-state connectivity, Demidenko et al. (2022) recently examined network heterogeneity during a reward task in a sample of older adolescents and early adults (mean age = 19 years). They identified two subgroups, one of which had increased connectivity paths in reward, cognitive control, and salience networks, and another which had fewer subgroup-level paths that were concentrated in cortical regions. Participants in the subgroup characterized by increased network density had increased self-reported sensation-seeking behavior. Within this subgroup, connectivity between the ventromedial prefrontal cortex and right orbitofrontal cortex was positively associated with sensation-seeking behavior and connectivity between the right orbitofrontal cortex and right ventral striatum was negatively associated with sensation-seeking behavior.

Studies have used GIMME to identify subsets of individuals more similar in their network functioning and have found differences between subgroups on measures of clinical functioning, increasing the ability to make inferences on an individual level. However, little work has been done assessing heterogeneity of networks at the group-level before subgroup identification, or examining network heterogeneity in adolescent samples. Here, we explore heterogeneity of reward networks in 103 early adolescents (mean age = 11.32 years, *SD* = 1.46) and examine associations between network features and behavioral outcomes, depression, and risk for alcohol and substance use problems. The study of reward networks in adolescence is an important extension as adolescence is a key risk period for the onset of multiple mental disorders such as depression (Costello et al., 2011; Solmi et al., 2021) and substance use problems (Grant & Dawson, 1998; Poudel & Gautam, 2017), which are in part characterized by alterations in reward functioning during adolescence (Casey et al., 2019; Forbes & Dahl, 2012; Heitzeg et al., 2015). First, we characterize qualitative network heterogeneity between individuals by examining whether an aggregate connectivity network is representative of connectivity networks identified in individuals. Second, we identify data-driven subgroups of individuals with similar reward networks, test the robustness of this solution, and examine if subgroup membership is associated with reward-related outcomes. Finally, we test the effect of network features on an individual level by examining associations between individualized connectivity paths and reward-related outcomes in the sample using regularized regressions.

## METHODS

The study was preregistered on Open Science Framework (https://doi.org/10.17605/osf.io/6n5j2). Deviations from the preregistration include the Early Adolescent Temperament Questionnaire pleasure intensity scale not being available from parent report and the additions of exploratory analyses examining network associations with risk for alcohol and substance use disorders and behavioral outcomes at 27-month follow-up after acquisition of supplemental funding. Time series data and open code for reproducing analyses are available on Open Science Framework (https://doi.org/10.17605/osf.io/7dgp4).

### Study Participants and Exclusion Criteria

Participants came from the Temple Adolescent Depression Study. The study was approved by Temple University’s Institutional Review Board (IRB no. 23174) and consent forms were signed prior to participation. Youth between ages 9 and 14 years with a primary caregiver were eligible for participation. Youth with a history of neurological disorder, head injury, pervasive developmental disorders, and/or intellectual functioning less than 70 as assessed by the Kaufman Brief Intelligence Test (Kaufman & Kaufman, 2013) were ineligible for study participation. Youth also were ineligible for participation if they had a history of bipolar disorder, psychosis spectrum disorders, developmental disorders or disabilities, neurological or cardiovascular diseases that affected central nervous system blood flow, were taking any psychotropic medications at the time of recruitment or scan, or were not able to complete an MRI scan safely. In total, 175 participants consented and completed the MRI portion of the study. Participants were excluded in a four-stage quality control process to reduce the effect of noise, particularly motion. First, participants were excluded if there were scanning issues (*N* = 5), incidental radiological findings (*N* = 1), or <75% behavioral compliance with the scanning task (*N* = 32). Second, participants were excluded based on manual inspection of MRIQC (Esteban et al., 2017) outputs for artifacts such as severe ringing or signal loss (*N* = 15). Third, participants were excluded after fmriprep preprocessing if their temporal signal-to-noise ratio (tSNR) or framewise displacement (FD) value was less than or greater than 1.5 times the interquartile range for each parameter, respectively (*N* = 5). Finally, participants were excluded if more than 25% of their frames had a FD value of at least 2 mm (*N* = 12). In total, 70 of the initial 175 participants were excluded, resulting in a sample of 105. The final sample had a mean age of 11.32 years (*SD* = 1.46), 58% were female, 48% were White, 36% were Black or African American, 1% were Asian, 11% were multiracial, and 4% preferred not to identify a race; 6% of the sample were Hispanic.

### fMRI Acquisition and Scanning Task

Neuroimaging data were acquired using a 3T Philips Ingenia scanner. BOLD functional images were acquired with a gradient echo planar imaging sequence and covered 34 axial slices (3 mm thick; TR = 2,000 ms, TE = 25 ms, field of view = 20 cm, matrix = 64 × 64).

We used a Card Guessing task (Forbes et al., 2006) that is frequently used in studies of monetary incentives (see Forbes et al., 2009, for task schematic). This event-related task examines responses to monetary gains and losses. Each trial includes both anticipation and outcome periods, and participants receive win, loss, or no-change feedback for each trial. Participants were told that their performance determines a monetary reward to be received after the scan, such that they would receive $1 for each win, lose 50 cents for each loss, and no change for neutral outcomes. Trials were presented in a pseudorandom order with predetermined outcomes. During each 17-second trial, participants had 4 seconds to select whether the value of a visually presented card with a possible value of 1–9 will be higher or lower than 5. After a choice was made, the trial type (reward or loss) is presented visually for 6 seconds (anticipation). The “actual” numerical value of the card is briefly displayed (500 ms), followed by outcome feedback (500ms), and, finally, a crosshair is presented for 7 seconds (outcome). The task included a jittered intertrial interval that averages 4 seconds. The task included 24 trials and lasted 8 minutes 2 seconds (239 acquisitions). The participants were unaware of the fixed outcome probabilities and were led to believe that outcomes were solely due to chance. The participants’ engagement and motivation were maintained by verbal encouragement during practice and between tasks in the magnet.

### Behavioral Measures

Several child-about-self and parent-about-child measures were examined as reward-related behavioral outcomes. Measures related to reward approach behaviors, inhibitory control, and clinical functioning and risk were selected as tests of external validity (i.e., whether individual differences in identified reward-related network features were associated with differences in reward-related behavioral outcomes). For parent report, we relied on reports from primary caretakers (99% mothers). When reports were unavailable for primary caretakers, we relied on reports from secondary caretakers. Correlations between behavioral measures are reported in the Supporting Information. Descriptive statistics of each behavioral measure for the included sample and excluded participants are provided in the Supporting Information.

#### Discounting rate.

The Delay Discounting task (McClure et al., 2004) assessed child preference for smaller, but immediate, rewards relative to larger, delayed rewards. Higher reward sensitivity reflects a greater preference for the immediate reward, indexed by the log of the discounting rate (log(k)). Participants chose between immediate and delayed rewards (e.g., $400 today or $1,000 in a week) considered roughly equal over four delay intervals (1 week, 1 month, 6 months, 1 year).

#### Early Adolescent Temperament Questionnaire (EATQ).

The EATQ (Ellis & Rothbart, 2001) is a 103-item self-report measure assessing temperament and self-regulation in children and adolescents. Items are rated on a five-point Likert scale, ranging from “Almost always untrue,” to “Almost always true.” EATQ Pleasure Sensitivity subscale contains seven items assessing pleasure related to activities or stimuli involving low intensity, rate, complexity, novelty, and incongruity. The Pleasure Sensitivity subscale is only assessed in the child self-report EATQ (α = .85, ω = .85). The EATQ Inhibitory Control subscale is included in the child self- and parent report EATQ. Inhibitory control represents the capacity to suppress inappropriate responses (youth α = 62, ω = .49; parent α = .64, ω = .60).

#### Pleasure Scale for Children (PSC).

The PSC (Kazdin, 1989) is a 39-item self-report measure assessing hedonic responses to rewarding events and activities in children. Youth were asked to indicate on a three-point Likert scale if that activity would make them feel “very happy,” “happy,” or if it “wouldn’t matter.” The PSC was only administered to child participants (α = .97, ω = .96).

#### Behavioral inhibition system/behavioral activation system (BIS/BAS).

The BIS/BAS (Carver & White, 1994) is a 24-item self-report measure assessing appetitive and aversive motivation. Participants rate each item on a four-point Likert scale ranging from “Very true for me” to “Very false for me.” We used the Drive, Reward Responsiveness, and Fun seeking subscales of the BAS. Both children and parents completed the BIS/BAS about the child. The BAS Drive subscale contains four items that reflect persistent pursuit of desired goals (youth α = .75, ω = .78; parent α = 86, ω = .81). The BAS Reward Responsiveness subscale contains five items that reflect positive responses to the occurrence or anticipation of reward (youth α = 82, ω = .75; parent α = .79, ω = .74). The BAS Fun Seeking subscale contains four items that reflect desire for new rewards and a willingness to approach a potentially rewarding event spuriously (youth α = .74, ω = .68; parent α = .72, ω = .68).

#### Child Depression Inventory (CDI).

The CDI (Kovacs, 1985) is a 27-item self-report measure assessing the cognitive, affective, and behavioral symptoms of depression in children and adolescents. Participants endorse statements about their experience of depressive symptoms on a three-point scale (youth α = .95, ω = .91; parent α = .92, ω = .90).

#### Mood and Feelings Questionnaire (MFQ).

The MFQ (Angold et al., 1995) is 33-item self-report measure assessing recent depressive symptoms. Participants endorse whether depressive symptoms are “Not True,” “Sometimes True,” or “True” of them in the past two weeks (youth α = .98, ω = .96; parent α = 95, ω = .94).

### Exploratory Measures

#### Alcohol expectations (AE) scale.

The AE scale contains 15 items that measure adolescent attitudes and behavioral expectations for alcohol use. Participants endorse degree of agreement with statements about expectations after drinking (e.g., “After a few drinks of alcohol, I would be more likely to be courageous/calm/sociable”) on a five-point Likert scale ranging from “Strongly Disagree” to “Strongly Agree.”

#### Familial risk for substance use problems.

Familial risk was assessed by maternal history of diagnosis of alcohol and/or substance use disorder was assessed by the Structured Clinical Interview for the DSM-5 (SCID-5; First et al., 2016).

#### Follow-up measures.

Follow-up measures of each of the above outcome variables (excluding familial risk) were assessed at a 27-month follow-up assessment. There was high attrition due to the COVID-19 pandemic. Follow-up sample sizes ranged from 41 (child-reported inhibitory control) to 59 (child-reported depression) and are fully described in the Supporting Information.

### fMRI Preprocessing and Time Series Extraction

Preprocessing was performed using fmriprep 20.2.6 (Esteban et al., 2019, 2022a; RRID:SCR_016216), which is based on Nipype 1.7.0 (Esteban et al., 2022b; Gorgolewski et al., 2011; RRID:SCR_002502). Fmriprep’s fieldmap-less distortion correction was used. BOLD runs were slice-time corrected with a middle slice reference and resampled onto the default MNI152NLin2009cAsym space. Full fmriprep preprocessing details are provided in the Supporting Information.

Further preprocessing was conducted in CONN toolbox, version 20.b (Whitfield-Gabrieli & Nieto-Castanon, 2012) in MATLAB 2017a. Minimally preprocessed data from fmriprep were spatially smooth with a 6-mm full-width at half-maximum Gaussian kernel. Data were then denoised using the first six aCompCor components, three translational motion regressors and their first derivatives, three rotational motion regressors and their first derivatives, the full set of cosine regressors, spike regressors defined by volumes with greater than 1.5-mm FD or 2.0 standardized DVARS values, and nonsteady-state outliers, as calculated by fmriprep. Time series were then extracted from 15 regions of interest (ROIs) using CONN’s default Harvard-Oxford probabilistic atlas (Desikan et al., 2006). The ROIs were the ACC, PCC, ventromedial prefrontal cortex (vmPFC; average of frontal medial cortex and subcallosal cortex), and the bilateral orbitofrontal cortex (OFC), insula, caudate, putamen, nucleus accumbens (NAcc), and amygdala.

### Analysis Plan

All statistical analyses on fMRI timeseries were conducted in R 4.0.1. Package versions are listed in the Supporting Information.

#### Effective connectivity network modeling.

We used several related iterative extended unified structural equation modeling (euSEM) processes to parse effective connectivity network heterogeneity at different levels of data aggregation. Effective connectivity is distinct from correlation-based functional connectivity in that it tests a directional model as the mechanism that generated the data, improving the ability to test hypotheses about how the brain is functioning (Friston, 2011). Effective connectivity modeling with euSEM differs from Dynamic Causal Modeling (DCM; Friston et al., 2003), a common approach to effective connectivity modeling, in two key ways. First, euSEM, as implemented here, is a data-driven approach, while DCM is confirmatory. Second, euSEM convolves activation with the hemodynamic response function, but does not assume relationships between neural and hemodynamic processes. However, several simulations have shown euSEM outperforming DCM in uncovering true network structures (Gates et al., 2011; Gates & Molenaar, 2012). For each network in this study, estimation begins with a null model, adds the contemporaneous, lagged, or autoregressive connectivity path that best improves model fit based on modification indices, until no further edges would do so. For all networks, nodes were the 15 ROIs and the reward anticipation and reward outcome exogenous task events that were convolved with the hemodynamic response function.

#### Aggregate and idiographic networks.

First, to assess how well a group-level model reflects each individual, we used euSEM to identify idiographic directed effective connectivity network models for the group aggregate and each individual (Gates et al., 2010, 2011). The aggregate group-level model was estimated using the *aggSEM* function in the GIMME package (Gates & Molenaar, 2012). This function concatenates time series from each participant into a single time series, then iteratively adds network paths that significantly improve model fit based on modification indices, beginning with an empty null model. To estimate idiographic network models, we used the *indSEM* function in the GIMME package in R. Using the same network identification process as the aggregate model, *indSEM* iteratively estimates networks for each individual independently, using no information from the group. We then examined how many paths of each individual-level network paths were present in the aggregate model.

#### Subgroup networks.

Second, we examined the potential presence of subgroups of individuals with more homogenous network features using GIMME (Gates et al., 2017). GIMME is an iterative model building application that relies on different levels of data aggregation. Simulations have shown that GIMME effectively detects more true edges and fewer spurious relationships than many other connectivity approaches, with the core distinction being that GIMME explicitly accounts for sample heterogeneity (Gates & Molenaar, 2012). Group-level network paths were first identified for the whole sample using paths that are significant for the majority of individuals (default 75%). Subgroups of individuals with similar network properties were then identified based on beta weights and modification indices of group-level paths, using a Walktrap community detection algorithm (Pons & Latapy, 2005). Subgroup-level paths were then iteratively added to each individual in the subgroup with a default majority threshold of >50%. Subgrouping GIMME has been shown to accurately capture subgroup classification in Monte Carlo simulations and improve the detection of the presence and direction of effects compared to the default GIMME process (Beltz & Gates, 2017).

To examine robustness of the identified subgroup solution, we used the *perturbR* package (Gates et al., 2019). Perturbr (1) assesses the stability of the subgrouping solution after iteratively randomly changing edges in the matrix and (2) compares the modularity of the obtained similarity matrix against simulated matrices to test if the obtained value is greater than what would be expected by chance. Similarity matrices are simulated by a weighted extension of the Erdos-Renyi binary random matrix approach which maintains statistical equivalence of nodes (i.e., ROIs), thereby maintaining the weighted properties of the original similarity matrix (Garlaschelli, 2009; Gates et al., 2019). The preregistered robustness criteria were (1) the similarity matrix requiring at least 20% of its edges being perturbed before 20% of individuals were placed in different clusters, and (2) the modularity for the original solution being greater than or equal to the 95th percentile of modularity obtained simulated matrices. A solution that passes these criteria will be regarded as robust to noise and modular (i.e., independent).

#### Individual networks.

Finally, as group-level information has been shown to improve detection of individual-level paths (Gates et al., 2017; Gates & Molenaar, 2012), we examined individualized networks estimated using the group-level model as the null. GIMME completes its model identification process by using the group and subgroup (if identified) networks as a null model for each individual, and then iteratively adds individual-level paths. Since the individual-level search follows the subgroup estimation process, we will only identify individual-level networks following the subgroup search if the subgroup solution passes the preregistered robustness criteria. If the subgroup solution does not pass the preregistered robustness criteria, we will estimate the individual-GIMME networks without a subgroup search.

#### Associations with behavioral outcomes.

After identifying connectivity network models, we examined how network features were associated with behavioral reward-related outcomes. To decrease the number of comparisons, we reduced the BAS subscales, PSC, and EATQ pleasure sensitivity subscale to a single reward sensitivity factor, and the CDI and MFQ to a depression aggregate score. These processes were completed separately for child- and parent-reported measures. Exploratory factor analysis (EFA) solutions for reward sensitivity are described in the Supporting Information. For depression, we standardized sum scores of the CDI and MFQ and averaged them. If one scale score was missing for a reporter, the other sum score was used by itself. Scores on the child- and parent-reported reward sensitivity factor, child- and parent-reported depression aggregate, child discounting rate, and child- and parent-reported inhibitory control were the final behavioral outcomes.

For idiographic and individual-GIMME models, we examined network features’ behavioral associations using adaptive lasso (Zou, 2006) in the *glmnet* R package. Adaptive lasso is an L1-regularized regression method that uses different regularization penalties for each coefficient. As coefficients are permitted to be penalized to 0, adaptive lasso also performs feature selection, a useful tool due to the large number of features provided by GIMME. Adaptive lasso provides a final set of features (i.e., directed connectivity paths) that are associated relevant outcome variable and their respective beta weights. Here, we followed a similar procedure as Dajani et al. (2020), but included binary features of whether a path was present or absent (i.e., statistically significant) for idiographic and individual-GIMME paths, rather than their beta weight, which would have been missing from individuals without that path. For both idiographic and individual-GIMME paths, paths were only considered if they were present for at least 20% of the sample (21 individuals). For GIMME group-level paths, beta weights were used since they are estimated for every individual. Adaptive penalties were determined by a ridge regression with 10-fold cross-validation, and adaptive lasso was then fit with a 10-fold cross-validation. All independent variables were standardized. We also calculated *R*^2^ and adjusted-*R*^2^ values for the final set of features for each outcome variable. Ultimately, this approach aims to identify what set of connectivity paths explain the maximum variance in a given behavioral outcome.

Behavioral differences between subgroups were tested using an analysis of variance (ANOVA) to examine omnibus differences with a Benjamini–Hochberg false discovery rate correction (Benjamini & Hochberg, 1995) to adjust for multiple comparisons. Post hoc pairwise comparisons examined differences between subgroups if omnibus tests were statistically significant.

## RESULTS

In initial GIMME models, path estimates for two participants yielded extreme outlier values. These two participants were removed, and all network models were reestimated with the remaining 103 participants.

### Idiographic and Aggregate Networks

**Figure 1.**
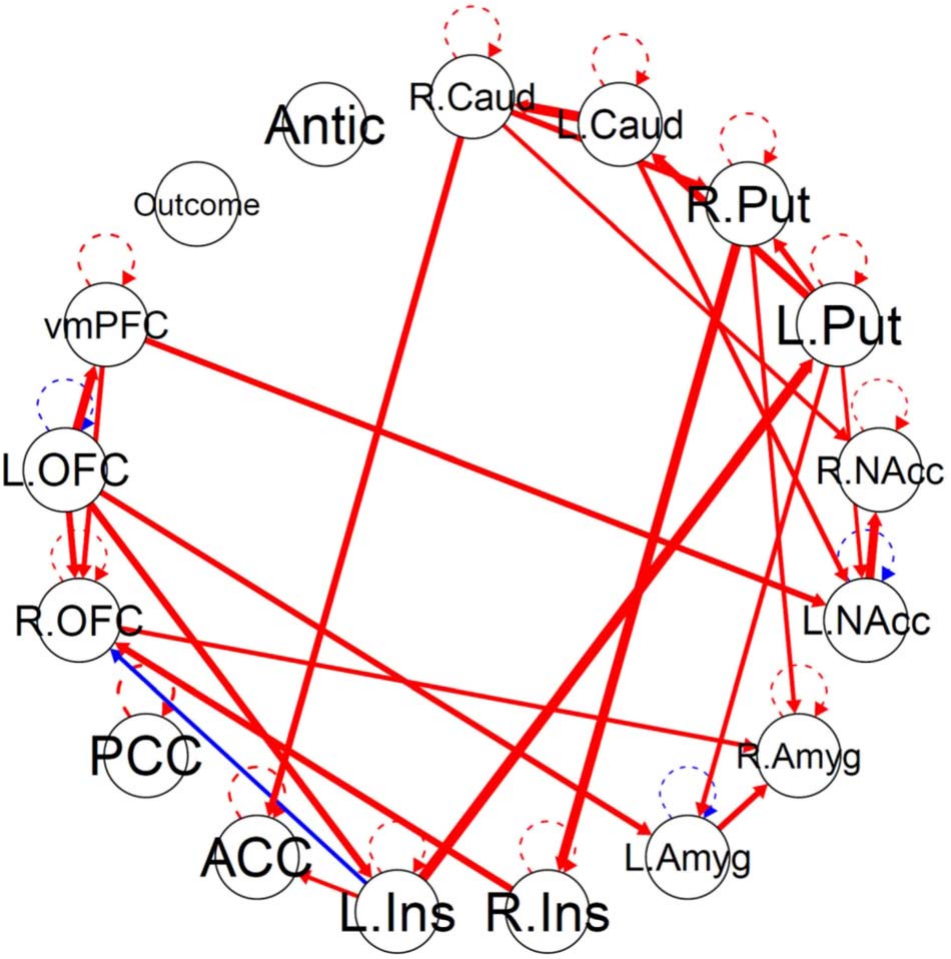
Aggregate connectivity network. Nodes are 15 ROIs and two exogenous task events convolved with hemodynamic response function. Red edges indicate significant positive connectivity paths for the averaged group. Blue edges indicate negative paths. Edge thickness corresponds with beta weight. L. = left. R. = right; Caud = caudate; Put = putamen; NAcc = nucleus accumbens. Amyg = amygdala; Ins = insula; ACC = anterior cingulate cortex; PCC = posterior cingulate cortex; OFC = orbitofrontal cortex; vmPFC = ventromedial prefrontal cortex; Antic = anticipation.

Figure 1 shows the aggregate directed connectivity network. Connectivity paths that were either significant in the aggregate network or at least 20% of the sample (21 individuals) idiographic networks are listed in the Supporting Information. The median idiographic network only shared 10 of the 24 aggregate paths (mean = 9.4) and 85 of the 103 participants (83%) shared less than half (<12) of the aggregate paths. Additionally, the two idiographic paths that were significant for the largest number of individuals were not significant in the aggregate network. The idiographic network most resembling the aggregate network had 17 of the 24 paths, and two idiographic networks had 16 of the 24 paths. Visual comparisons between the aggregate model, the closest resembling individual model, the median-resembling individual model, and the least-resembling individual model are shown in the Supporting Information. Overall, the aggregate level network was not a good representation of individual-level networks.

### Subgrouping GIMME

**Figure 2.**
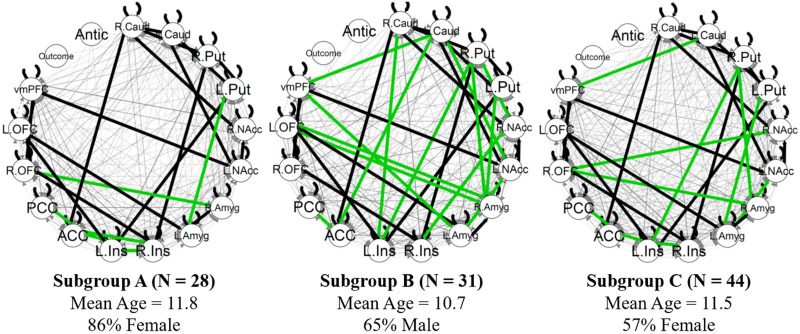
GIMME subgroups. Black edges reflect connectivity paths significant for the group (same for all subgroups). Green edges reflect subgroup-specific connectivity paths. Gray edges reflect individual-level paths identified after the subgroup search. L. = left; R. = right; Caud = caudate; Put = putamen; NAcc = nucleus accumbens; Amyg = amygdala; Ins = insula; ACC = anterior cingulate cortex; PCC = posterior cingulate cortex; OFC = orbitofrontal cortex; vmPFC = ventromedial prefrontal cortex; Antic = anticipation.

GIMME identified three subgroups (Figure 2). Subgroup characteristics are described in Table 1. Subgroup 1 has the least dense connectivity network; it has four cortical paths between the bilateral insular regions, the ACC, and the PCC, and two subcortical paths, one from the left putamen to the left amygdala and another from the right OFC to the right amygdala. Participants in Subgroup 2 had the densest connectivity network, with 13 of the 14 subgroup-level paths involving subcortical regions. Participants in Subgroup 3 had an intermediately dense network, with 6 of the 10 paths involving striatal regions. Participants in Subgroup 1were significantly older than participants in other subgroups and were predominantly female. Participants in Subgroup 2 were mostly male and were significantly younger than participants in Subgroup 1, but not significantly younger than participants in Subgroup 3. Subgroup 3 had the most participants, had a near even split between sexes, and had participants that were significantly older than those in Subgroup 2, but not significantly different in age than participants in Subgroup 1. The subgroups appear to reflect reward network maturity, with more mature subgroups having denser networks, older participants, and predominantly female participants.

**Table 1.** GIMME subgroup characteristics

	Subgroup 1	Subgroup 2	Subgroup 3	Omnibus test
Number of individuals	28	31	44	–
Number of subgroup paths	6	14	10	–
Age mean (*SD*)	11.76 (1.53)_a_	10.73 (1.32)_b_	11.49 (1.40)_b_	*F*(2,98) = 4.18*
Percent female (no.)	86% (24)_a_	35% (11)_b_	57% (25)_c_	χ^2^(2) = 16.36**
Framewise displacement in mm (*SD*)	0.27 (0.13)_a_	1.23 (0.50)_b_	0.62 (0.34)_c_	*F*(2,98) = 26.13**

*Note*. For each row, subgroups that do not share the same subscript are different at *p* < .05.

* *p* < .05.

** *p* < .001.

In tests of the preregistered subgroup validity criteria, the obtained subgroup solution was stable (i.e., robust to noise), passing the first criterion, but had low modularity, failing the second criterion. Using the Variation of Information criteria (see Supporting Information), approximately 35% edges had to be perturbed before 20% of participants were placed into different clusters. Additionally, according to the adjusted Rand index, the solutions when 20% of edges were perturbed were more similar to the original model than a model with 20% of cluster assignments randomly swapped (*t* = 102, *p* < .001). These indices suggest that the Walktrap clustering algorithm reliably produced the same subgrouping solution as noise iteratively increased, until about one third of paths were perturbed. However, the subgroup solution had very low modularity (.03). Furthermore, sensitivity analyses indicated that participants in different subgroups significantly differed in scanner movement, raising the possibility that subgroups were identified based on movement, rather than distinct network properties.

Overall, the obtained subgroups were robust to noise, but have very low modularity. Thus, we present the subgroup solution and examine differences between subgroups on reward outcomes, as previous studies have solely utilized robustness to noise to assess validity of GIMME subgroups (Kaurin et al., 2022). However, as the subgroup solution did not pass our preregistered modularity criteria, we also estimate GIMME without subgroups for examination of individual-level paths.

### Individual GIMME

**Figure 3.**
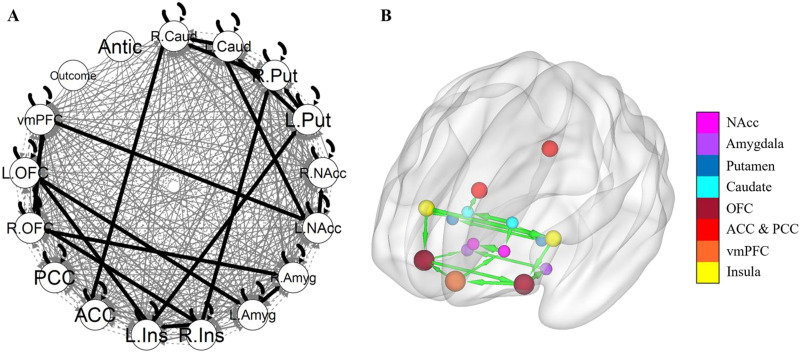
GIMME group network. (A) The GIMME output network, with black edges representing group-level paths and gray edges representing individual-level paths. (B) The same group-level paths projected onto a smoothed MNI glass brain. Edge thickness corresponds to the path’s beta weight. The brain network was visualized with the BrainNet Viewer (Xia et al., 2013). L. = Left. R; = Right; Caud = caudate; Put = putamen; NAcc = nucleus accumbens; Amyg = amygdala; Ins = insula; ACC = anterior cingulate cortex; PCC = posterior cingulate cortex; OFC = orbitofrontal cortex; vmPFC = ventromedial prefrontal cortex; Antic = anticipation.

Figure 3 shows the GIMME group-level network without a subgroup search. There were 19 group-level paths (Table 2). The paths with the strongest weights, excluding lateralized paths of same region, were from the left insula to the left putamen, from the right insula to the right putamen, from the left OFC to the left amygdala, from the left putamen to the left caudate, and from the left OFC to the vmPFC. GIMME identified five group-level paths that were not identified in the aggregate model: from the right insula to the right putamen, from the left insula to the left OFC, from the right insula to the left insula, from the right putamen to the right caudate, and from the right OFC to the left OFC. Conversely, the aggregate group model identified 10 paths that were not present in the GIMME group model. No group-level paths involving the reward outcome or anticipation were identified.

**Table 2.** GIMME group-level paths

**From**	**To**	**Beta (*SD*)**
Left insula	Left putamen	0.77 (0.19)
Left caudate	Right caudate	0.63 (0.22)
Right insula	Right putamen	0.59 (0.20)
Left orbitofrontal cortex	Ventromedial prefrontal cortex	0.52 (0.33)
Ventromedial prefrontal cortex	Left nucleus accumbens	0.40 (0.20)
Left nucleus accumbens	Right nucleus accumbens	0.66 (0.22)
Ventromedial prefrontal cortex	Right orbitofrontal cortex	0.38 (0.21)
Left putamen	Left caudate	0.57 (0.32)
Left orbitofrontal cortex	Left amygdala	0.59 (0.60)
Right insula	Right orbitofrontal cortex	0.44 (0.20)
Right caudate	Anterior cingulate cortex	0.42 (0.25)
Right orbitofrontal cortex	Right amygdala	0.30 (0.24)
Left insula	Left orbitofrontal cortex	0.49 (0.20)
Left caudate	Left nucleus accumbens	0.36 (0.23)
Left putamen	Right putamen	0.45 (0.23)
Left amygdala	Right amygdala	0.46 (0.24)
Right insula	Left insula	0.66 (0.66)
Right putamen	Right caudate	0.32 (0.23)
Right orbitofrontal cortex	Left orbitofrontal cortex	0.50 (0.30)

*Note*. Paths are listed in the order identified iteratively by GIMME, using modification indices.

GIMME networks fit the data well for the majority of participants. Using fit criteria of comparative fit index (CFI) ≥ .90, nonnormal fit index (NNFI) ≥ .90, root-mean-square error of approximation (RMSEA) ≤ .08, and standardized root-mean-squared residual (SRMR) ≤ .08, 100 participants met all four criteria, 1 participant met two of the four criteria, and 2 participants did not meet any of the criteria. Individual models from GIMME also demonstrated substantial network heterogeneity, such that the presence of various individual-level paths differed widely across adolescents. Table 3 lists the 17 individual-level paths that were significant for at least 21 participants. The most common individual-level paths were from the right caudate to the right NAcc and from the right putamen to the right amygdala. A visualization of differences in individual-level paths across select participants is presented in the Supporting Information. We also estimated GIMME models without including exogenous task regressors, and the resulting models were nearly identical. All GIMME outputs are provided on OSF (osf.io/zymq5).

**Table 3.** GIMME individual-level paths

**From**	**To**	**Count**
Right caudate	Right nucleus accumbens	41
Right putamen	Right amygdala	37
Right insula	Left OFC	31
Left insula	Right putamen	30
Posterior cingulate cortex	Anterior cingulate cortex	28
Left insula	Left amygdala	27
Left insula	Anterior cingulate cortex	26
Left putamen	Left amygdala	26
Anterior cingulate cortex	Posterior cingulate cortex	23
Left amygdala	Left nucleus accumbens	23
Left putamen	Left nucleus accumbens	22
Right caudate	Left insula	22
vmPFC	Anterior cingulate cortex	22
Right insula	Right amygdala	21
Right putamen	Left insula	21
Right caudate	Left caudate	21
Right nucleus accumbens	Right insula	21

*Note*. Count of the number of individuals that the path was statistically significant. Paths only listed if significant in at least 20% of sample (>21 individuals).

### Associations With Behavioral Outcomes

There were no significant differences in any of the preregistered outcomes—discounting rate, reward sensitivity, inhibitory control, and depression—across subgroups. The two participants that did not meet two out of four fit criteria were excluded from further analyses. Table 4 displays results of associations between GIMME network features and behavioral outcomes using adaptive lasso. Network paths were associated with age, sex, discounting rate, child- and parent-reported reward sensitivity, and child- and parent-reported inhibitory control, and child-reported depression. Adaptive lasso did not identify any paths that were associated with parent-reported depression. Based on *R*^2^, network features were most associated with child-reported reward sensitivity (adjusted *R*^2^ = .19, 10 paths) and parent-reported inhibitory control (adjusted *R*^2^ = .16, 7 paths). The individual-level path from the vmPFC to the ACC was associated with the greatest number of outcomes, including discounting rate, child-reported reward sensitivity-child report, and parent-reported inhibitory control. Sensitivity analyses accounting for scanner movement were conducted by assessing associations after inclusion of mean FD for each participant. Results were largely similar after including mean FD. Full results are presented in the Supporting Information.

**Table 4.** GIMME network associations with behavioral outcomes

Outcome	From	To	Beta	Path level	Total *R*^2^	Adjusted *R*^2^
Age	Left nucleus Accumbens	Right nucleus Accumbens	−0.17	Group	0.22	0.16
	Ventromedial prefrontal cortex	Right orbitofrontal cortex	−0.12	Group		
	Right caudate	Anterior cingulate cortex	−0.20	Group		
	Right insula	Left insula	0.10	Group		
	Right caudate	Left caudate	−0.02	Indiv		
	Right putamen	Right amygdala	−0.22	Indiv		
	Left putamen	Left nucleus accumbens	−0.13	Indiv		
Sex	Right insula	Right putamen	0.32	Group	0.16	0.13
	Left Insula	Left orbitofrontal cortex	0.17	Group		
	Right orbitofrontal cortex	Left orbitofrontal cortex	0.26	Group		
	Left putamen	Left nucleus accumbens	0.27	Indiv		
Discounting rate	Left orbitofrontal cortex	Ventromedial prefrontal cortex	0.67	Group	0.16	0.14
	Right putamen	Left insula	0.15	Indiv		
	Ventromedial prefrontal cortex	Anterior cingulate cortex	0.22	Indiv		
Reward sensitivity (child report)	Left orbitofrontal cortex	Ventromedial prefrontal cortex	0.09	Group	0.27	0.19
	Right insula	Right orbitofrontal cortex	−0.08	Group		
	Right caudate	Anterior cingulate cortex	0.03	Group		
	Left insula	Left orbitofrontal cortex	−0.09	Group		
	Right orbitofrontal cortex	Left orbitofrontal cortex	0.21	Group		
	Right caudate	Left caudate	0.01	Indiv		
	Right nucleus accumbens	Right insula	0.17	Indiv		
	Left insula	Anterior cingulate cortex	−0.10	Indiv		
	Posterior cingulate cortex	Anterior cingulate cortex	−0.16	Indiv		
	Ventromedial Prefrontal cortex	Anterior cingulate cortex	−0.24	Indiv		
Reward sensitivity (parent report)	Left insula	Left putamen	0.08	Group	0.08	0.05
	Right insula	Right putamen	0.05	Group		
	Right insula	Right amygdala	−0.08	Indiv		
Inhibitory control (child report)	Right caudate	Left insula	−1.01	Indiv	0.10	0.07
	Left insula	Anterior cingulate cortex	0.42	Indiv		
	Posterior cingulate cortex	Anterior cingulate cortex	−0.19	Indiv		
Inhibitory control (parent report)	Left insula	Left putamen	−0.51	Group	0.22	0.16
	Ventromedial prefrontal cortex	Right orbitofrontal cortex	−0.21	Group		
	Left caudate	Left nucleus accumbens	0.36	Group		
	Left amygdala	Right amygdala	0.16	Group		
	Right caudate	Left caudate	−0.28	Indiv		
	Anterior Cingulate cortex	Posterior cingulate cortex	−0.59	Indiv		
	Ventromedial prefrontal cortex	Anterior cingulate cortex	−0.20	Indiv		
Depression (child report)	Left amygdala	Right amygdala	−0.04	Group	.02	0.01
Depression (parent report)	–	–	–	–	–	–
Baseline alcohol expectancies*	–	–	–	–	–	–
Follow-up alcohol expectancies*	Right orbitofrontal cortex	Left orbitofrontal cortex	0.42	Group	0.27	0.23
	Right putamen	Left insula	0.09	Indiv		
	Left amygdala	Left nucleus accumbens	−3.02	Indiv		
	Right insula	Right amygdala	1.45	Indiv		
	Anterior cingulate cortex	Posterior cingulate cortex	−3.24	Indiv		
Familial risk for substance use problems*	Ventromedial prefrontal cortex	Right orbitofrontal cortex	0.59	Group	.40	.31
	Left insula	Left orbitofrontal cortex	−0.46	Group		
	Right insula	Left insula	−0.34	Group		
	Right putamen	Right caudate	−0.59	Group		
	Right caudate	Left caudate	−0.34	Indiv		
	Right caudate	Right nucleus accumbens	−0.58	Indiv		
	Right caudate	Left insula	−0.55	Indiv		
	Right insula	Left orbitofrontal cortex	0.26	Indiv		
	Left insula	Right putamen	0.20	Indiv		
	Left insula	Left amygdala	0.24	Indiv		
	Left insula	Anterior cingulate cortex	−0.24	Indiv		
	Anterior cingulate cortex	Posterior cingulate cortex	−0.25	Indiv		
	Posterior cingulate cortex	Anterior cingulate cortex	0.30	Indiv		

*Note*. Positive relationship with sex indicates association with being male. Group-level features used each participant’s beta weight. Individual-level features used binary significance of that path for each individual. Indiv = Individual.

* Post hoc test.

Adaptive lasso results using idiographic network features are presented in the Supporting Information. Results and overall trends were similar to those using GIMME networks, although the number of significant features and the strength of associations tended to be higher using idiographic network features.

### Post Hoc Analyses

The final adaptive lasso model explained 31% of the variance in having parental history of alcohol and/or substance use disorder using 13 paths. The group-level path from the ventromedial prefrontal cortex to the right orbitofrontal cortex and the individual-level paths from the right putamen to the right caudate and from the right caudate to the right nucleus accumbens had the strongest associations. No paths were associated with child alcohol expectancies at baseline. Since participants at baseline may be too young (mean age = 11.32) for alcohol expectancies, we also explored associations with alcohol expectancies at 27-month follow-up with adaptive lasso. The final model explained 23% of the variance in alcohol expectancies at follow-up using five paths. The individual-level paths from the left amygdala to the left NAcc and from the ACC to the PCC had the strongest associations.

As split-sample testing was not used, we also conducted post hoc linear regressions with each selected feature to obtain estimates of standard error and significance tests. Although adaptive lasso feature selection is not a test of statistical significance, a large number of selected features had significant associations in the linear regressions. Adjusted *R*^2^ values also tended to be higher in the linear regression models. Full results are presented in the Supporting Information.

Follow-up analyses examined whether identified baseline features for each measure continued to be significantly associated with the respective measure at 27-month follow-up. We tested the significance of each of the baseline features for the respective 27-month measure using a general linear model. Follow-up child-reported reward sensitivity was significantly associated with the significance of the right NAcc to right insula path at baseline. Additionally, follow-up parent-reported inhibitory control was significantly associated with the significance of the ACC to PCC path at baseline. These associations were no longer significant after controlling for the false discovery with a Benjamini–Hochberg correction (Benjamini & Hochberg, 1995). No other baseline features were significantly associated with respective measures at 27-month follow-up.

## DISCUSSION

This study examined qualitative reward network functioning between early adolescents and tested the relationship between individualized reward network features and reward-related behavioral outcomes, depression, and risk for substance use disorder. Results showed substantial heterogeneity in reward network function between adolescents, indicating that a group-level model was not representative of individuals. Connectivity paths between striatal and prefrontal regions were associated with multiple behavioral outcomes and had the strongest relationships with child-reported reward sensitivity, parent-reported inhibitory control, and familial history of alcohol and/or substance use disorder. These findings caution the reliance on group aggregate networks for studying behavioral phenotypes such as clinical disorders and suggest a viable alternative in the focus of individualized network features.

The majority of individual-level networks shared less than half of the connectivity paths that were significant in the group aggregate model, indicating that the aggregate model was a poor representation of individuals. This model nonconformity prevents generalizing findings from group-level analyses to individuals (Molenaar, 2004). This substantially diminishes the clinical utility of network neuroscience findings as individual-level inferences are not valid. Moreover, implications extend beyond clinical use of fMRI to basic research, where group averages may obscure individual differences in qualitative network functioning during a task that reflect distinct underlying psychological processes. While this result is concerning for a field that largely relies on group averages, it is consistent with previous findings that individuals exhibit traitlike network features that are not captured in an aggregate network (Gratton et al., 2020; Seitzman et al., 2019). See Medaglia et al. (2011) for a discussion of nonergodicity in network neuroscience and Fisher et al. (2018) for a discussion of the threat of nonergodicity in psychological processes more broadly.

An increasingly common approach to examine heterogeneity in network function is the identification of subgroups of individuals who are more homogeneous in their network functioning. Subgrouping approaches capture multidimensional information in a single categorical variable, rather than interactions between all ROIs that would require very large samples (Feczko et al., 2019). Here, we used Walktrap community detection in GIMME to identify three distinct subgroups. The subgroups reflected differences in reward network maturity, as they differed in reward network density, age, and sex. However, the subgroups do not resemble those previously identified by Demidenko et al. (2022), who used GIMME to examine reward network functioning in a sample of older adolescents and early adults. Differences in identified subgroups may be due to differences between the studies in the developmental stages, ROIs, atlases, and tasks. The Card Guessing task used here is a reward decision-making task, and the monetary incentive delay task used by Demidenko et al. (2022) is an instrumental reward task (Richards et al., 2013).

Furthermore, while unsupervised clustering methods will identify a solution, the internal and external validity of the solution must be examined to determine utility (Brucar et al., 2023). Here, we found modest evidence of subgroup validity. Testing internal validity, the identified subgroups do not appear to be a result of noise, as they showed robustness to increased levels of path perturbations. However, the subgroups had low modularity, suggesting high overlap between them, likely due to the large number of group-level paths. Additionally, the subgroups significantly differed in mean FD, leaving uncertainty to whether they capture differences in reward network maturity, movement, or both. Testing external validity, individuals across subgroups did not significantly differ in reward-related behavior outcomes. Together, these results highlight the importance of assessing both internal validity, such as robustness to noise, differences in movement, and solution modularity, as well as external validity such as differences in behavioral outcomes when using data-driven network subgroup approaches.

The group-level GIMME network contained 19 total connectivity paths, which largely consisted of ipsilateral connections between subcortical reward-related regions and prefrontal regions, as well as connections between homologous regions across hemispheres. This is a denser group-level network than that identified in Demidenko et al. (2022), potentially due to this sample being younger, as adolescence is characterized by a shift from diffuse connectivity to stronger, focalized connectivity networks (Khundrakpam et al., 2016). Shared group-level paths between the two studies include those from the right OFC to the right amygdala, from the left amygdala to the right amygdala, and from the right OFC to the left OFC.

In contrast to a subgroup-level focus, analyses focusing on the external utility of *individualized* connectivity paths to behavioral outcomes showed promise. We assessed relationships between individual-level network features and behavioral outcomes using adaptive lasso in two ways. For group-level paths that were significant for at least 75% of individuals, we tested associations between individual differences in connectivity strength and behavioral outcomes. For individual-level connectivity paths, we tested whether a path being significant in each individual was associated with behavioral outcomes. Importantly, this approach focused analyses on network features that are precise to each individual, rather than a group average. Using adaptive lasso, a regularized regression technique that performs feature selection and penalized coefficient estimation specific to the sample, we found that individualized features were associated with multiple reward-related functions and risk for substance use problems, but not with depressive symptoms. Consistent with previous research on delay discounting (Anandakumar et al., 2018), we found that connectivity between regions involved with cognitive control regions was associated with discounting rate. We also observed a pattern where increased group-level connectivity and number of individual-level connections between subcortical to cortical regions were associated with child-reported increased reward sensitivity and decreased inhibitory control, while increased connectivity/number of connections between cortical regions were associated with decreased reward sensitivity and increased inhibitory control. These results are consistent with previous findings of separate systems for adolescent reward functioning and cognitive control (Casey et al., 2008).

Clinically, network features were associated with parental history of alcohol and/or substance use disorder, a key risk factor for future adolescent substance use problems (Hoffmann & Cerbone, 2002). The relationships were characterized by decreased connectivity between several subcortical regions and the insula, and increased connectivity between the vmPFC and right OFC. These results are consistent with previous findings that adolescents with familial risk for alcohol and substance use problems have decreased connectivity in frontostriatal regions (for reviews, see Cservenka, 2016, for alcohol and Heitzeg et al., 2015, and Squeglia & Cservenka, 2017, for substance use). Results suggest particular relevance of individualized reward network functioning in the risk for adolescent substance use problems. Furthermore, the utility of individualized network features here provides further support for an increased focus on precision functional connectivity estimation, rather than group-level estimation (Gratton et al., 2020). In contrast, although previous studies have found associations between reward-related brain function and depression (Forbes & Dahl, 2012), we did not find robust associations. It is possible that brain-behavior associations in the early adolescence sample used here are more specific to reward-based behavioral dimensions (e.g., discounting rate, reward sensitivity) or specific clinical symptoms (e.g., anhedonia), rather than aggregate symptom scores.

### Strengths and Limitations

Strengths of this study include preregistration, open data and code, examination of the internal validity of the subgrouping solution, and assessment of multiple distinct child- and parent-reported reward-related behaviors and clinical outcomes. This study also had several limitations that future research should address. First, the length of the scan may limit power to reliably detect network structures at the individual level. Precision imaging studies have suggested that scans may require more than 40-minutes to reliably estimate individual’s networks (i.e., retest reliability > 0.9; Gratton et al., 2020), although other work has demonstrated reliability of individual network differences in shorter scan lengths (Birn et al., 2013; Duda et al., 2023). Furthermore, poor reliability of individual-level networks has downstream consequences on the reliability of associations with individual differences in behavioral outcomes. GIMME partially mitigates this concern by estimating individual-level features after identifying group-level features, and simulations have shown strong reliability in detecting individual-level features (Beltz & Gates, 2017; Gates & Molenaar, 2012). Future work should emphasize scans with more measures to increase reliability of network estimation and brain-behavior associations. Similarly, longer scans with more task effects can increase power to detect effects of exogenous events on connectivity, which was low here (discussed in more detail in Demidenko et al., 2022). Second, the sample size was not large enough to use training and testing samples to assess reliability of the subgrouping solution in external data or the ability of networks paths to *predict* behavioral outcomes. As such, associations identified by adaptive lasso may be overfit to the sample used here. Research with larger sample sizes, such as in the Adolescent Brain Cognitive Development (ABCD) study (Casey et al., 2018) or Human Connectome Project (HCP; Van Essen et al., 2013) can address these limitations going forward by testing for prediction in external samples and examining potential model overfit. There was also particularly low power to detect behavioral associations with follow-up measurements due to attrition related to COVID-19. Third, while the exclusion criteria were selected to reduce noise in the BOLD signal, it altered the representativeness of the sample’s age due to increased head motion (see Supporting Information). Finally, the vector autoregression approach that GIMME uses assumes that network edges are consistent across the time series (Gates et al., 2011). However, this assumption may conflict with connectivity at different stages of the task used in this study (i.e., win, loss, neutral changes) or in other GIMME studies (e.g., Dajani et al., 2020; Demidenko et al., 2022; McCormick et al., 2019). Further work with precision imaging approaches may increase the power to model multiple exogenous task events to help address this limitation.

### Conclusion

Overall, our results show substantial qualitative heterogeneity in reward networks across early adolescents such that an aggregate model does not adequately represent individual-level models. These findings suggest that group-average comparisons may have limited utility to examine associations between network functioning and behavioral and clinical outcomes, as findings may not be generalizable to the individual. While previous studies have identified subgroups of distinct network processes and detected clinical differences between them, we found little validity in a subgrouping solution and no behavioral differences between subgroups. In contrast, individualized network features were associated with multiple reward-related behavioral outcomes and risk for substance use problems, showing promise for detecting brain-behavior relationships specific to the individual.

## ACKNOWLEDGMENTS

Training in GIMME methodology was gained through Dr. Kathleen Gates’s workshop at the University of Pittsburgh’s Summer Methodology Series.

## SUPPORTING INFORMATION

Supporting information for this article is available at https://doi.org/10.1162/netn_a_00306.

## AUTHOR CONTRIBUTIONS

Matthew Mattoni: Conceptualization; Data curation; Formal analysis; Funding acquisition; Investigation; Methodology; Project administration; Visualization; Writing – Original draft; Writing – Review & editing. David V. Smith: Methodology; Supervision; Writing – Review & editing. Thomas M. Olino: Conceptualization; Formal analysis; Funding acquisition; Methodology; Project administration; Supervision; Writing – review & editing.

## FUNDING INFORMATION

Thomas M. Olino, National Institute of Mental Health (https://dx.doi.org/10.13039/100000025), Award ID: R01 MH107495. Matthew Mattoni, Weinstein Family/Civic Foundation Award. Publication of this article was funded in part by the Temple University Libraries Open Access Publishing Fund.

## TECHNICAL TERMS

Reward network: Connectivity between brain regions associated with approach motivation, wanting, and positive emotions related to anticipating or consuming a reward.
Heterogeneity: Qualitative differences in connectivity network patterns that are not attributable to random error and result in distinct network structures.
Effective connectivity: Hypothesized underlying directional network that models how activation in one region influences others to explain the observed correlations in BOLD activity.
Ergodicity: The ability to generalize group-level statistics (e.g., averages) to individuals.
euSEM: Approach to effective connectivity that models contemporaneous and lagged relationships between nodes, as well as effects of exogenous variables.
Functional connectivity: Correlation between BOLD activity of two brain regions or voxels.
Idiographic: Study of a single individual (*N* = 1) rather than a group.
Adaptive lasso: Regression method where coefficients are differentially penalized to reduce bias. Penalties can minimize coefficients to 0, performing feature selection.
