# Supplementary material for: Characterizing heterogeneity in early adolescent reward networks and individualized associations with behavioral and clinical outcomes
Source: Netw Neurosci. 2023 Jun 30;7(2):787–810. doi: 10.1162/netn_a_00306 (PMC10312268; doi:10.1162/netn_a_00306)
Supplement: Supplementary file 1 [file netn-7-2-787-s001.pdf]

Mattoni, M., Smith, D. V. & Olino, T. M. (2023). Supporting information for "Characterizing Heterogeneity in Early Adolescent Reward Networks and Individualized Associations with Behavioral and Clinical Outcomes." *Network Neuroscience*. Advance publication. https://doi.org/10.1162/netn_a_00306

Characterizing Heterogeneity in Early Adolescent Reward Networks and Individualized Associations with Behavioral and Clinical Outcomes

Supplementary Materials

Table of Contents

**S1. Brief Literature Review**	2-3
**S2. R Packages**	3-4
**S3. Follow-Up Sample Sizes**	4
**S4. fmriprep details**	5-7
**S5. Reward Drive EFA**	8
**S6. Behavioral Correlations**	9-10
**S7. Idiographic and Aggregate Paths & Visual Comparison**	11-12
**S8. Subgroup Validation**	13
**S9. Heterogeneity Demonstration**	14
**S10. FD Sensitivity Analysis**	15-16
**S11. Idiographic Network Behavioral Associations**	17-19
**S12. Linear Regression Results**	19-20
**S13. Descriptive Statistics of Behavioral Variables**	21
**References**	22-23

S1. Brief Review of Relevant Connectivity Network Subgrouping Studies

The majority of work taking a subgrouping approach has been done in adult samples and using resting state networks. In one of the largest studies, Drysdale et al. (2017) used canonical correlation analysis and hierarchical clustering to identify subgroups of resting state connectivity networks associated with depression. They found a subgroup defined by hypoconnectivity in frontoamygdala and orbitofrontal networks, a subgroup defined by hypoconnectivity in anterior cingulate and orbitofrontal networks, a subgroup defined by hyperconnectivity in thalamic and frontostriatal networks, and a subgroup defined by hypoconnectivity just in frontoamygdala regions. They further found clinical utility, as individuals in the subgroup with hypoconnectivity in anterior cingulate and orbitofrontal networks had decreased symptom severity while individuals in the subgroup with hypoconnectivity in frontoamygdala and orbitofrontal networks had greater responses to treatment with transcranial magnetic stimulation.

Liang et al. (2020) used k-means clustering in another large sample of depressed patients and found two subgroups with distinct default mode network (DMN) connectivity profiles; one subgroup had hypoconnectivity in the DMN, while the other subgroup had hyperconnectivity in the DMN, particularly in the prefrontal regions. However, individuals in the two subgroups did not significantly differ on depression severity or symptoms. In one of the few studies of neural network heterogeneity in children or adolescents, Costa Dias et al. (2015) used community detection analysis on resting state networks to identify three subgroups with distinct reward connectivity profiles. They further found that in different subgroups differed by behavioral impulsivity, and that associations between certain connectivity paths and Attention-Deficit/Hyperactivity Disorder (ADHD) diagnosis differed across subgroups. Kashyap et al. (2020) used common-and-orthogonal-basis-extraction similarity and dissimilarity to identify two subgroups of resting state connectivity in the human connectome project. They found the subgroup defined by decreased default mode network (DMN) connectivity had higher rates of alcohol and illicit substance use than a more homogenous subgroup characterized by strong DMN connectivity. Finally, Zhu et al. (2022) used k-means and hierarchical clustering to identify three subgroups of resting state connectivity in a sample of adults from the human connectome project with at least one symptom of alcohol use disorder. The three subgroups

corresponded to individuals with mild symptoms, moderate symptoms, and symptoms comorbid with antisocial personality disorder. The mild-symptom subgroup was characterized by increased cerebellar and prefrontal connectivity but decreased cerebellar and temporal pole connectivity, the moderate-symptom subgroup was characterized by the densest network and increased connectivity between the insula and supramarginal gyrus, and the comorbid subgroup was characterized by increased connectivity between motor and temporal regions.

S2. R Packages

Package	Version	Citation
base	4.0.1	R Core Team (2020)
gimme	0.7.5	Lane et al. (2021)
glmnet	4.1.3	Friedman, Hastie, and Tibshirani (2010); Simon et al. (2011)
grateful	0.1.11	Rodríguez-Sánchez, Jackson, and Hutchins (2022)
lavaan	0.6.10	Rosseel (2012)
perturbR	0.1.3	Gates, Fisher, and Arizmendi (2019)
psych	2.1.9	Revelle (2021)
renv	0.15.2	Ushey (2022)
tidyverse	1.3.0	Wickham et al. (2019)

We used R version 4.0.1 (R Core Team 2020) and the following R packages: gimme v. 0.7.5 (Lane et al. 2021), glmnet v. 4.1.3 (Friedman, Hastie, and Tibshirani 2010; Simon et al. 2011), grateful v. 0.1.11 (Rodríguez-Sánchez, Jackson, and Hutchins 2022), lavaan v. 0.6.10 (Rosseel 2012), perturbR v. 0.1.3 (Gates, Fisher, and Arizmendi 2019), psych v. 2.1.9 (Revelle 2021), renv v. 0.15.2 (Ushey 2022), tidyverse v. 1.3.0 (Wickham et al. 2019).

Package citations

Friedman, Jerome, Trevor Hastie, and Robert Tibshirani. 2010. "Regularization Paths for Generalized Linear Models via Coordinate Descent." *Journal of Statistical Software* 33 (1): 1–22. https://www.jstatsoft.org/v33/i01/.

Gates, KM, Zachary Fisher, and Cara Arizmendi. 2019. *PerturbR: Random Perturbation of Count Matrices*. https://CRAN.R-project.org/package=perturbR.

Lane, Stephanie, Kathleen Gates, Zachary Fisher, Cara Arizmendi, Peter Molenaar, Michael Hallquist, Hallie Pike, et al. 2021. *Gimme: Group Iterative Multiple Model Estimation*. https://CRAN.R-project.org/package=gimme.

R Core Team. 2020. *R: A Language and Environment for Statistical Computing*. Vienna, Austria: R Foundation for Statistical Computing. https://www.R-project.org/.

Revelle, William. 2021. *Psych: Procedures for Psychological, Psychometric, and Personality Research*. Evanston, Illinois: Northwestern University. https://CRAN.R-project.org/package=psych.

Rodríguez-Sánchez, Francisco, Connor P. Jackson, and Shaurita D. Hutchins. 2022. *Grateful: Facilitate Citation of R Packages*. https://github.com/Pakillo/grateful.

Rosseel, Yves. 2012. "lavaan: An R Package for Structural Equation Modeling." *Journal of Statistical Software* 48 (2): 1–36. https://doi.org/10.18637/jss.v048.i02.

Simon, Noah, Jerome Friedman, Trevor Hastie, and Rob Tibshirani. 2011. "Regularization Paths for Cox's Proportional Hazards Model via Coordinate Descent." *Journal of Statistical Software* 39 (5): 1–13. https://www.jstatsoft.org/v39/i05/.

Ushey, Kevin. 2022. *Renv: Project Environments*. https://CRAN.R-project.org/package=renv.

Wickham, Hadley, Mara Averick, Jennifer Bryan, Winston Chang, Lucy D'Agostino McGowan, Romain François, Garrett Grolemund, et al. 2019. "Welcome to the tidyverse." *Journal of Open Source Software* 4 (43): 1686. https://doi.org/10.21105/joss.01686.

S3. Follow-Up Sample Sizes

Follow-up was measured 27 months after the initial scan. There was high attrition due to the COVID-19 pandemic. Parent-reported depression and child-reported alcohol expectancy scores were not tested as there were no significant predictors at baseline.

Measure	Sample Size
Discounting Rate	58
Reward Sensitivity (Child Report)	57
Reward Sensitivity (Parent Report)	54
Inhibitory Control (Child Report)	41
Inhibitory Control (Parent Report)	46
Depression (Child Report)	59

S4. fMRI Preprocessing

Anatomical data preprocessing

A total of 1 T1-weighted (T1w) images were found within the input BIDS dataset. The T1-weighted (T1w) image was corrected for intensity non-uniformity (INU) with N4BiasFieldCorrection (Tustison et al. 2010), distributed with ANTs 2.3.3 (Avants et al. 2008, RRID:SCR_004757), and used as T1w-reference throughout the workflow. The T1w-reference was then skull-stripped with a Nipype implementation of the antsBrainExtraction.sh workflow (from ANTs), using OASIS30ANTs as target template. Brain tissue segmentation of cerebrospinal fluid (CSF), white-matter (WM) and gray-matter (GM) was performed on the brain-extracted T1w using fast (FSL 5.0.9, RRID:SCR_002823, Zhang, Brady, and Smith 2001). Volume-based spatial normalization to one standard space (MNI152NLin2009cAsym) was performed through nonlinear registration with antsRegistration (ANTs 2.3.3), using brain-extracted versions of both T1w reference and the T1w template. The following template was selected for spatial normalization: ICBM 152 Nonlinear Asymmetrical template version 2009c [Fonov et al. (2009), RRID:SCR_008796; TemplateFlow ID: MNI152NLin2009cAsym],

Functional data preprocessing

For each subject's BOLD run, the following preprocessing was performed. First, a reference volume and its skull-stripped version were generated using a custom methodology of fMRIPrep. A deformation field to correct for susceptibility distortions was estimated based on fMRIPrep's fieldmap-less approach. The deformation field is that resulting from co-registering the BOLD reference to the same-subject T1w-reference with its intensity inverted (Wang et al. 2017; Huntenburg 2014). Registration is performed with antsRegistration (ANTs 2.3.3), and the process regularized by constraining deformation to be nonzero only along the phase-encoding direction, and modulated with an average fieldmap template (Treiber et al. 2016). Based on the estimated susceptibility distortion, a corrected EPI (echo-planar imaging) reference was calculated for a more accurate co-registration with the anatomical reference. The BOLD reference was then co-registered to the T1w reference using flirt (FSL 5.0.9, Jenkinson and Smith 2001) with the boundary-based registration (Greve and Fischl 2009) cost-function. Co-registration was configured with nine degrees of freedom to account for distortions remaining in

the BOLD reference. Head-motion parameters with respect to the BOLD reference (transformation matrices, and six corresponding rotation and translation parameters) are estimated before any spatiotemporal filtering using mcflirt (FSL 5.0.9, Jenkinson et al. 2002). BOLD runs were slice-time corrected to 0.975s (0.5 of slice acquisition range 0s-1.95s) using 3dTshift from AFNI 20160207 (Cox and Hyde 1997, RRID:SCR_005927). The BOLD time-series (including slice-timing correction when applied) were resampled onto their original, native space by applying a single, composite transform to correct for head-motion and susceptibility distortions. These resampled BOLD time-series will be referred to as preprocessed BOLD in original space, or just preprocessed BOLD. The BOLD time-series were resampled into standard space, generating a preprocessed BOLD run in MNI152NLin2009cAsym space. First, a reference volume and its skull-stripped version were generated using a custom methodology of fMRIPrep. Several confounding time-series were calculated based on the preprocessed BOLD: framewise displacement (FD), DVARS and three region-wise global signals. FD was computed using two formulations following Power (absolute sum of relative motions, Power et al. (2014)) and Jenkinson (relative root mean square displacement between affines, Jenkinson et al. (2002)). FD and DVARS are calculated for each functional run, both using their implementations in Nipype (following the definitions by Power et al. 2014). The three global signals are extracted within the CSF, the WM, and the whole-brain masks. Additionally, a set of physiological regressors were extracted to allow for component-based noise correction (CompCor, Behzadi et al. 2007). Principal components are estimated after high-pass filtering the preprocessed BOLD time-series (using a discrete cosine filter with 128s cut-off) for the two CompCor variants: temporal (tCompCor) and anatomical (aCompCor). tCompCor components are then calculated from the top 2% variable voxels within the brain mask. For aCompCor, three probabilistic masks (CSF, WM and combined CSF+WM) are generated in anatomical space. The implementation differs from that of Behzadi et al. in that instead of eroding the masks by 2 pixels on BOLD space, the aCompCor masks are subtracted a mask of pixels that likely contain a volume fraction of GM. This mask is obtained by thresholding the corresponding partial volume map at 0.05, and it ensures components are not extracted from voxels containing a minimal fraction of GM. Finally, these masks are resampled into BOLD space and binarized by thresholding at 0.99 (as in the original implementation). Components are also calculated separately within the WM and CSF masks. For each CompCor decomposition, the k components with the largest singular

values are retained, such that the retained components' time series are sufficient to explain 50 percent of variance across the nuisance mask (CSF, WM, combined, or temporal). The remaining components are dropped from consideration. The head-motion estimates calculated in the correction step were also placed within the corresponding confounds file. The confound time series derived from head motion estimates and global signals were expanded with the inclusion of temporal derivatives and quadratic terms for each (Satterthwaite et al. 2013). Frames that exceeded a threshold of 1.5 mm FD or 2 standardised DVARS were annotated as motion outliers. All resamplings can be performed with a single interpolation step by composing all the pertinent transformations (i.e. head-motion transform matrices, susceptibility distortion correction when available, and co-registrations to anatomical and output spaces). Gridded (volumetric) resamplings were performed using antsApplyTransforms (ANTs), configured with Lanczos interpolation to minimize the smoothing effects of other kernels (Lanczos 1964). Non-gridded (surface) resamplings were performed using mri_vol2surf (FreeSurfer).

Many internal operations of fMRIPrep use Nilearn 0.6.2 (Abraham et al. 2014, RRID:SCR_001362), mostly within the functional processing workflow. For more details of the pipeline, see the section corresponding to workflows in fMRIPrep's documentation.

S5. Reward Drive EFA

For child-report, we conducted an EFA in R using indicators of BAS Drive, BAS Reward Responsiveness, BAS Fun Seeking, Pleasure Scale for Children, and EATQ Fun Seeking. Kaiser–Meyer–Olkin values were near .7 for all items, suggesting data are appropriate for a factor analysis. We used an oblimin rotation and a maximum likelihood estimator. Velicer's MAP first suggested a 1 or 2 factor solution. Factors in the 2-factor solution had a correlation of .51, suggesting high similarity. Additionally, all items loaded on the single factor solution with loadings greater than .30. We selected the single factor solution for parsimony. Factor loadings are presented in the table below.

Scale Score	1-Factor Solution – Loading	2-Factor Solution – F1 Loading	2-Factor Solution – F2 Loading
BAS Drive	.49	.70	-.22
BAS Reward Responsiveness	.81	.63	.22
BAS Fun Seeking	.78	.77	.10
Pleasure Scale for Children	.62	.04	.92
EATQ Pleasure Sensitivity	.32	-.03	.46

For parent-report, the PSC and EATQ Pleasure Sensitivity scales are unavailable, so only BAS Drive, Reward Responsivity, and Fun Seeking scales were used. Kaiser–Meyer–Olkin values were near .7 for all items, suggesting data are appropriate for a factor analysis. Velicer's MAP first suggested a 1 or 2 factor solution, and parallel analysis with 1000 iterations suggested a 1 factor solution. Each BAS scale had a loading greater than .75 on the single factor solution, so it was selected. Drive had a loading of .87, Reward Responsivity had a loading of .76, and Fun Seeking had a loading of .81.

Scale Score	1-Factor Solution – F1 Loading	2-Factor Solution – F1 Loading	2-Factor Solution – F2 Loading
BAS Drive	.87	.87	-.05
BAS Reward Responsiveness	.76	.76	-.03
BAS Fun Seeking	.81	.81	.09

S6. Behavioral Variable Correlations

S6.1 Correlations Between Raw Variables

	**1.**	**2.**	**3.**	**4.**	**5.**	**6.**	**7.**	**8.**	**9.**	**10.**	**11.**	**12.**	**13.**	**14.**	**15.**	**16.**
**1.**	1															
**2.**	0.66	1														
**3.**	0.7	0.62	1													
**4.**	-0.28	-0.17	-0.27	1												
**5.**	0.10	0.09	-0.06	-0.36	1											
**6.**	0.05	0.00	-0.12	-0.41	0.75	1										
**7.**	0.29	0.01	0.26	-0.12	-0.05	0.11	1									
**8.**	0.13	0.28	0.14	-0.06	-0.12	-0.05	0.40	1								
**9.**	0.32	0.29	0.39	-0.2	-0.03	0.04	0.47	0.62	1							
**10.**	0.02	0.22	0.11	-0.12	-0.09	-0.02	0.15	0.52	0.48	1						
**11.**	0.13	0.03	0.12	-0.12	0.13	0.14	0.08	0.03	0.00	0.12	1					
**12.**	-0.16	-0.01	-0.11	0.10	-0.06	-0.13	0.07	0.26	0.17	0.42	0.02	1				
**13.**	-0.38	-0.22	-0.23	0.40	-0.30	-0.37	-0.23	-0.1	-0.35	-0.05	-0.04	0.07	1			
**14.**	-0.04	-0.08	-0.14	-0.14	0.36	0.40	0.13	0.03	0.03	0.12	0.15	0.05	-0.35	1		
**15.**	0.03	-0.03	-0.14	-0.19	0.47	0.48	0.03	-0.14	-0.11	-0.01	0.13	0.02	-0.36	0.86	1	
**16.**	0.04	0.08	-0.04	-0.03	0.34	0.22	-0.03	0.00	0.06	-0.07	0.25	0.00	-0.16	0.06	0.13	1

1. BAS Drive (Parent) 2. BAS Reward Responsiveness (Parent) 3. BAS Fun Seeking (Parent) 4. EATQ Inhibitory Control (Parent) 5. MFQ (Parent) 6. CDI (Parent) 7. BAS Drive (Child) 8. BAS Reward Responsiveness (Child) 9. BAS Fun Seeking (Child) 10. Pleasure Scale for Children 11. Delay Discounting 12. EATQ Pleasure Sensitivity (Child) 13. EATQ Inhibitory Control (Child) 14. MFQ (Child) 15. CDI (Child) 16. Familial Risk

S6.2 Correlations between Aggregate Variables

	**1.**	**2.**	**3.**	**4.**	**5.**	**6.**	**7.**	**8.**
**1.**	1	0.11	0.16	-0.03	-0.14	0.09	0.1	0.25
**2.**	0.11	1	0.24	-0.23	-0.12	0.06	0.05	0.01
**3.**	0.16	0.24	1	-0.35	-0.27	-0.01	0.1	0.03
**4.**	-0.03	-0.23	-0.35	1	0.43	-0.38	-0.39	-0.16
**5.**	-0.14	-0.12	-0.27	0.43	1	-0.21	-0.49	-0.03
**6.**	0.09	0.06	-0.01	-0.38	-0.21	1	0.42	0.1
**7.**	0.1	0.05	0.1	-0.39	-0.49	0.42	1	0.3
**8.**	0.25	0.01	0.03	-0.16	-0.03	0.1	0.3	1

1. Delay Discounting 2. Reward Drive (Child) 3. Reward Drive (Parent) 4. Inhibitory Control (Child) 5. Inhibitory Control (Parent) 6. Depression (Child) 7. Depression (Parent) 8. Familial Risk

S7. Idiographic and Aggregate Paths

From	To	Count	In Aggregate?
Right Insula	Right Putamen	66	N*
Right Nucleus Accumbens	Left Nucleus Accumbens	62	N*
Right Insula	Right Orbitofrontal Cortex	62	Y
Left Insula	Left Putamen	57	Y
Left Insula	Left Orbitofrontal Cortex	56	N*
Ventromedial Prefrontal Cortex	Left Orbitofrontal Cortex	56	N*
Left Caudate	Right Caudate	54	Y
Right Caudate	Anterior Cingulate Cortex	53	Y
Left Putamen	Left Insula	53	N*
Left Amygdala	Right Amygdala	53	Y
Right Caudate	Left Caudate	51	N*
Left Putamen	Right Putamen	50	Y
Left Nucleus Accumbens	Right Nucleus Accumbens	49	Y
Left Orbitofrontal Cortex	Ventromedial Prefrontal Cortex	49	Y
Right Putamen	Right Caudate	48	N*
Right Orbitofrontal Cortex	Right Amygdala	48	Y
Left Orbitofrontal Cortex	Left Amygdala	48	Y
Right Putamen	Right Insula	46	Y
Left Orbitofrontal Cortex	Right Orbitofrontal Cortex	46	Y
Left Putamen	Left Caudate	45	Y
Ventromedial Prefrontal Cortex	Right Orbitofrontal Cortex	45	Y
Right Amygdala	Left Amygdala	44	N*
Left Insula	Right Insula	41	N
Right Orbitofrontal Cortex	Ventromedial Prefrontal Cortex	40	N*
Right Orbitofrontal Cortex	Left Orbitofrontal Cortex	37	N*
Right Putamen	Right Amygdala	36	Y
Left Caudate	Left Nucleus Accumbens	35	Y
Left Caudate	Left Putamen	34	N*
Right Orbitofrontal Cortex	Right Insula	33	N*
Ventromedial Prefrontal Cortex	Left Nucleus Accumbens	33	Y
Right Caudate	Right Putamen	32	Y
Right Caudate	Right Nucleus Accumbens	32	Y
Left Nucleus Accumbens	Left Caudate	32	N*
Right Putamen	Left Putamen	31	N*
Right Insula	Right Amygdala	31	N
Anterior Cingulate Cortex	Posterior Cingulate Cortex	31	N
Left Insula	Left Amygdala	28	N
Left Nucleus Accumbens	Ventromedial Prefrontal Cortex	27	N*
Right Insula	Left Insula	27	N
Left Orbitofrontal Cortex	Left Insula	27	Y
Left Amygdala	Left Insula	26	N
Left Amygdala	Left Orbitofrontal Cortex	26	N*
Ventromedial Prefrontal Cortex	Right Nucleus Accumbens	26	N
Left Caudate	Anterior Cingulate Cortex	25	N
Right Insula	Anterior Cingulate Cortex	25	N
Right Amygdala	Right Orbitofrontal Cortex	23	N*

Left Orbitofrontal Cortex	Left Nucleus Accumbens	23	N
Left Putamen	Left Amygdala	22	Y
Left Insula	Anterior Cingulate Cortex	<21	Y
Left Insula	Right Orbitofrontal Cortex	<21	Y
Left Putamen	Left Nucleus Accumbens	<21	Y

Count indicates the number of participants who had a significant connectivity path between the identified regions in their idiographic model. Y indicates that the path was significant in the aggregate model, N indicates that it was not significant, and N* indicates that it was not significant, but its reverse direction was.

Visual Aggregate-Individual Model Comparison

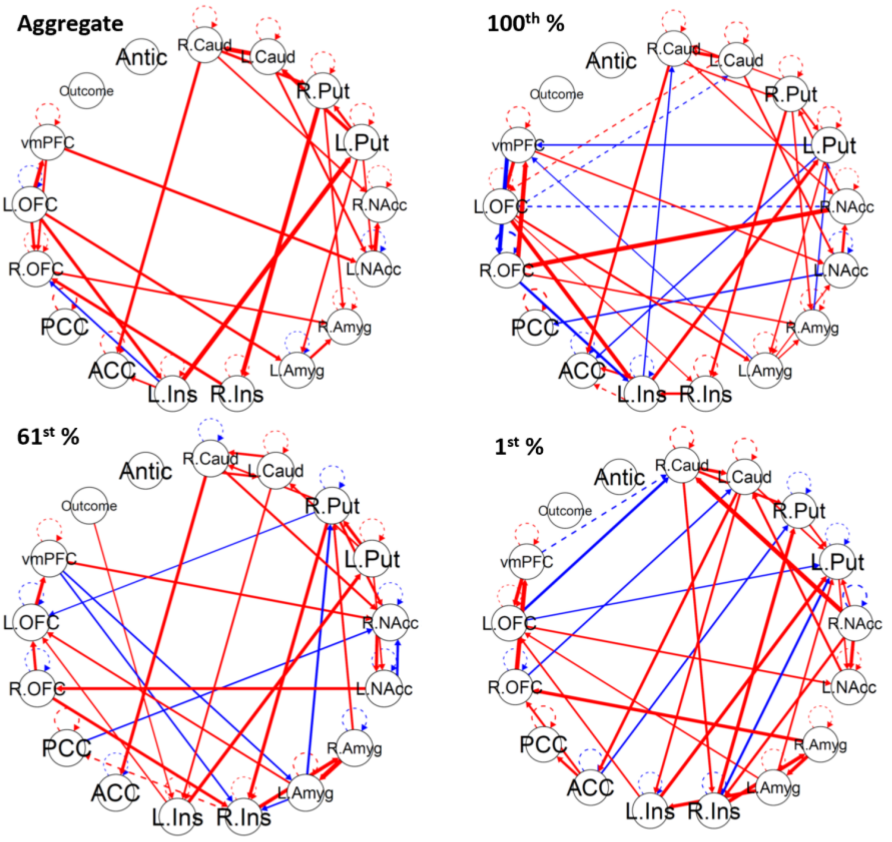

% = percentile. The closest-resembling network was subject-51, with 17 of the 24 aggregate paths. The closest median percentile was 61, with 10 of the 24 aggregate paths. Multiple subjects had 10 of the 24 paths, subject-01 is displayed here. The least-resembling model was subject-36, who had only 3 of the 24 aggregate paths.

S8. Subgroup Validation

Variation of Information Index

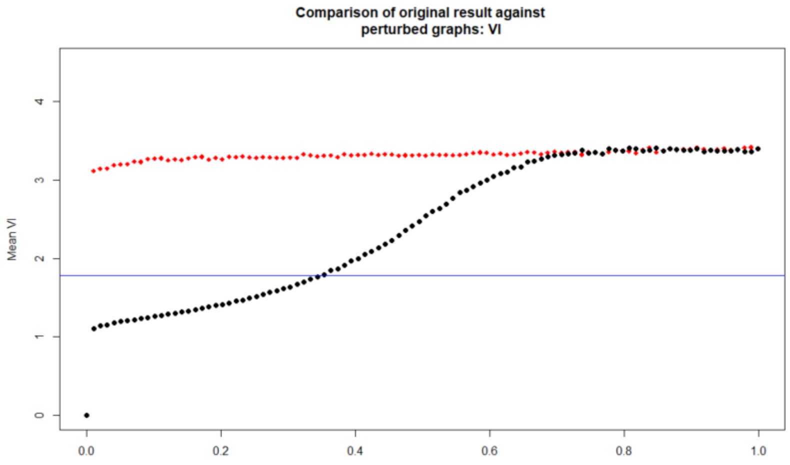

The x-axis is the proportion of perturbed edges. Horizontal Line represents point at which 20% of nodes are randomly swapped between clusters. Black dots represent perturbed paths based on original subgroup solution. Red dots represent the perturbed solution. The figure indicates that, based on VI, greater than 20% of edges (~35%) had to be perturbed before 20% of participants were placed into different clusters.

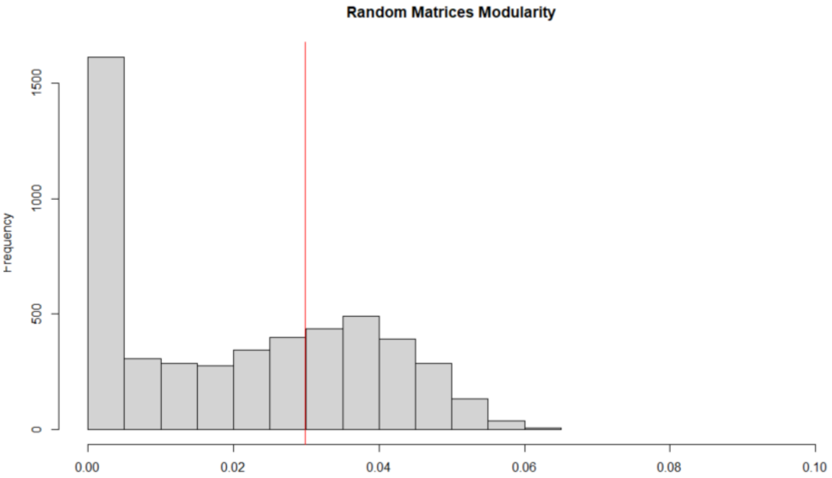

Figure above represents frequency of modularity values obtained in 5,000 iterations of the *perturbR* function.

S9. Individual-Level Path Heterogeneity

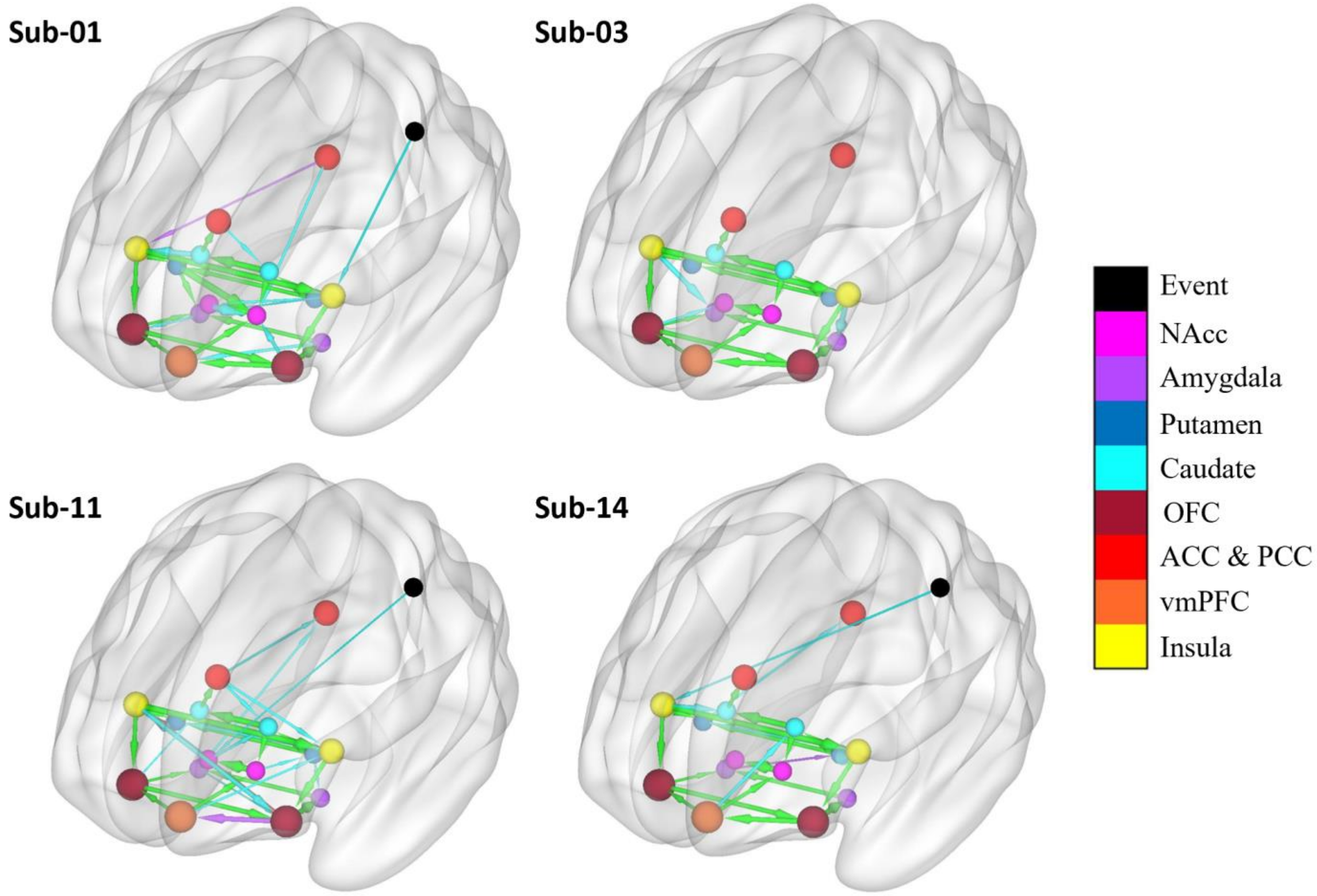

GIMME paths projected onto a smoothed MNI glass brain. Green paths are group-level, blue paths are contemporaneous individual-level, and purple paths are lagged individual-level. Edge thickness corresponds to the path's beta weight for group-level paths. Event for Sub-01 is reward outcome. Event for Sub-11 and Sub-14 is reward anticipation. Brain networks were visualized with the BrainNet Viewer (Xia et al., 2013).

S10. Framewise Displacement Sensitivity Analysis

Outcome	From	To	Beta	Path Level	Total R^2	Adjusted R^2
Age	Left Nucleus Accumbens	Right Nucleus Accumbens	-0.10	Group	0.17	0.11
	Ventromedial Prefrontal Cortex	Right Orbitofrontal Cortex	-0.02	Group		
	Right Caudate	Anterior Cingulate Cortex	-0.09	Group		
	Right Insula	Left Insula	.005	Group		
	Right Putamen	Right Amygdala	-0.16	Indiv		
	Left Putamen	Left Nucleus Accumbens	-0.04	Indiv		
	Framewise Displacement	-	-0.22	-		
Sex	Right Insula	Right Putamen	0.21	Group	0.17	0.13
	Left Insula	Left Orbitofrontal Cortex	0.09	Group		
	Right Orbitofrontal Cortex	Left Orbitofrontal Cortex	0.29	Group		
	Left Putamen	Left Nucleus Accumbens	0.23	Indiv		
	Framewise Displacement	-	0.22	-		
Delay Discounting	Left Orbitofrontal Cortex	Ventromedial Prefrontal Cortex	0.67	Group	0.16	0.14
	Right Putamen	Left Insula	0.16	Indiv		
	Ventromedial Prefrontal Cortex	Anterior Cingulate Cortex	0.22	Indiv		
Reward Drive (Child Report)	Left Orbitofrontal Cortex	Ventromedial Prefrontal Cortex	0.09	Group	0.27	0.19
	Right Insula	Right Orbitofrontal Cortex	-0.08	Group		
	Right Caudate	Anterior Cingulate Cortex	0.03	Group		
	Left Insula	Left Orbitofrontal Cortex	-0.09	Group		
	Right Orbitofrontal Cortex	Left Orbitofrontal Cortex	0.21	Group		
	Right Caudate	Left Caudate	0.01	Indiv		
	Right Nucleus Accumbens	Right Insula	0.17	Indiv		
	Left Insula	Anterior Cingulate Cortex	-0.10	Indiv		

	Posterior Cingulate Cortex	Anterior Cingulate Cortex	-0.16	Indiv		
	Ventromedial Prefrontal Cortex	Anterior Cingulate Cortex	-0.24	Indiv		
Reward Drive (Parent Report)	Right Insula	Right Putamen	0.05	Group	.08	.05
	Left Insula	Left Putamen	0.08	Group		
	Right Insula	Right Amygdala	-0.08	Indiv		
Inhibitory Control (Child Report)	Right Caudate	Left Insula	-1.01	Indiv	0.10	0.07
	Left Insula	Anterior Cingulate Cortex	0.42	Indiv		
	Posterior Cingulate Cortex	Anterior Cingulate Cortex	-0.19	Indiv		
Inhibitory Control (Parent Report)	Left Insula	Left Putamen	-3.38	Group	0.28	0.20
	Ventromedial Prefrontal Cortex	Left Nucleus Accumbens	-0.04	Group		
	Ventromedial Prefrontal Cortex	Right Orbitofrontal Cortex	-1.94	Group		
	Right Orbitofrontal Cortex	Right Amygdala	-0.84	Group		
	Left Insula	Left Orbitofrontal Cortex	1.18	Group		
	Left Caudate	Left Nucleus Accumbens	2.08	Group		
	Left Amygdala	Right Amygdala	1.28	Group		
	Right Caudate	Left Caudate	-0.68	Indiv		
	Anterior Cingulate Cortex	Posterior Cingulate Cortex	-1.48	Indiv		
	Ventromedial Prefrontal Cortex	Anterior Cingulate Cortex	-0.45	Indiv		
Depression (Child Report)	Left Insula	Left Putamen	-0.02	Group	0.03	0.01
	Left Amygdala	Right Amygdala	-0.23	Group		
Depression (Parent Report)	-	-	-	-	-	-

Indiv = Individual. Positive relationship with sex indicates association with being male. Group-level path predictors used each participant’s beta weight. Individual-level path predictors used binary significance of that path for each individual. Coefficient values are not comparable to original results as connectivity estimates were standardized with age.

S11. Idiographic Network Behavioral Associations from Adaptive Lasso

Outcome	From	To	Estimate	Total R^2	Adjusted R^2
Age	Right Caudate	Left Caudate	0.07	0.24	0.16
	Right Caudate	Anterior Cingulate Cortex	-0.26		
	Right Putamen	Right Amygdala	-0.24		
	Left Putamen	Right Putamen	0.14		
	Left Putamen	Left Amygdala	0.09		
	Left Nucleus Accumbens	Ventromedial Prefrontal Cortex	-0.22		
	Right Amygdala	Right Orbitofrontal Cortex	0.18		
	Right Insula	Right Orbitofrontal Cortex	0.09		
	Left Insula	Left Amygdala	-0.13		
	Anterior Cingulate Cortex	Posterior Cingulate Cortex	-0.04		
Sex	Right Putamen	Right Insula	-0.07	0.18	0.12
	Left Nucleus Accumbens	Right Nucleus Accumbens	0.03		
	Right Insula	Right Putamen	0.41		
	Right Insula	Right Orbitofrontal Cortex	-0.32		
	Left Insula	Right Insula	-0.07		
	Ventromedial Prefrontal Cortex	Left Orbitofrontal Cortex	0.07		
Delay Discounting	Left Amygdala	Left Orbitofrontal Cortex	0.18	0.08	0.04
	Right Insula	Left Insula	0.25		
	Anterior Cingulate Cortex	Posterior Cingulate Cortex	-0.07		
	Right Orbitofrontal Cortex	Ventromedial Prefrontal Cortex	-0.23		
Reward Sensitivity (Child Report)	Left Caudate	Anterior Cingulate Cortex	-0.05	0.27	0.17
	Right Putamen	Right Amygdala	0.14		
	Left Putamen	Left Caudate	0.12		
	Left Putamen	Left Amygdala	-0.09		
	Left Amygdala	Left Insula	0.13		
	Left Insula	Right Insula	-0.05		
	Right Orbitofrontal Cortex	Right Amygdala	-0.14		

	Right Orbitofrontal Cortex	Right Insula	-0.002		
	Right Orbitofrontal Cortex	Left Orbitofrontal Cortex	0.03		
	Right Orbitofrontal Cortex	Ventromedial Prefrontal Cortex	-0.24		
	Left Orbitofrontal Cortex	Left Amygdala	-0.04		
Reward Sensitivity (Parent Report)	Right Caudate	Left Caudate	0.05	0.23	0.14
	Right Putamen	Right Insula	0.09		
	Left Nucleus Accumbens	Ventromedial Prefrontal Cortex	0.14		
	Right Amygdala	Right Orbitofrontal Cortex	-0.04		
	Left Amygdala	Left Orbitofrontal Cortex	-0.02		
	Right Insula	Anterior Cingulate Cortex	-0.11		
	Left Insula	Left Putamen	0.06		
	Left Insula	Right Insula	-0.06		
	Left Insula	Left Orbitofrontal Cortex	-0.12		
	Right Orbitofrontal Cortex	Ventromedial Prefrontal Cortex	-0.16		
Inhibition (Child Report)	Left Caudate	Anterior Cingulate Cortex	0.70	0.16	0.13
	Left Putamen	Left Caudate	-0.26		
	Left Putamen	Left Amygdala	0.62		
	Ventromedial Prefrontal Cortex	Left Nucleus Accumbens	1.07		
Inhibition (Parent Report)	Right Caudate	Left Caudate	-0.33	0.47	0.34
	Right Caudate	Right Nucleus Accumbens	0.16		
	Right Caudate	Anterior Cingulate Cortex	-0.47		
	Left Caudate	Anterior Cingulate Cortex	0.48		
	Right Putamen	Left Putamen	0.10		
	Left Putamen	Left Caudate	-0.54		
	Right Amygdala	Left Amygdala	-0.11		
	Right Insula	Right Putamen	0.08		
	Right Insula	Right Amygdala	0.26		
	Left Insula	Left Putamen	-0.34		
	Left Insula	Left Orbitofrontal Cortex	0.30		

	Anterior Cingulate Cortex	Posterior Cingulate Cortex	-0.41		
	Right Orbitofrontal Cortex	Left Orbitofrontal Cortex	-0.33		
	Right Orbitofrontal Cortex	Ventromedial Prefrontal Cortex	0.47		
	Left Orbitofrontal Cortex	Left Amygdala	0.04		
	Left Orbitofrontal Cortex	Left Insula	-0.25		
	Left Orbitofrontal Cortex	Right Orbitofrontal Cortex	0.14		
	Left Orbitofrontal Cortex	Ventromedial Prefrontal Cortex	1.14		
	Ventromedial Prefrontal Cortex	Left Nucleus Accumbens	0.52		
	Ventromedial Prefrontal Cortex	Right Orbitofrontal Cortex	0.52		
	Ventromedial Prefrontal Cortex	Left Orbitofrontal Cortex	-0.15		
Depression (Child Report)	-		-	0	0
Depression (Parent Report)	Ventromedial Prefrontal Cortex	Left Nucleus Accumbens	-0.14	0.07	0.06

S12. Linear Regressions for Features Identified by Adaptive Lasso

Construct	Path	Beta (SE)	Total Adjusted R^2
Delay Discounting	L.OFC-vmPFC	0.99 (0.25)***	0.21
	R.Put-L.Ins	0.68 (0.25)**	
	vmPFC-ACC	0.64 (0.25)*	
Reward Sensitivity (Child Report)	L.OFC-vmPFC	0.13 (0.11)	0.22
	R.Ins-R.OFC	-0.15 (0.12)	
	R.Caud-ACC	0.09 (0.13)	
	L.Ins-L.OFC	-0.26 (0.11)*	
	R.OFC-L.OFC	0.26 (0.11)*	
	R.Caud-L.Caud	0.09 (0.12)	
	R.NAcc-R.Ins	0.24 (0.12)*	
	L.Ins-ACC	-0.20 (0.11)	
	PCC-ACC	-0.21 (0.11)	
	vmPFC-ACC	-0.30 (0.12)*	

Reward Sensitivity (Parent Report)	L.Ins-L.Put	0.26 (0.11)*	0.14
	R.Ins-R.Put	0.24 (0.11)*	
	R.Ins-R.Amyg	-0.28 (0.11)*	
Inhibitory Control (Child Report)	R.Caud-L.Ins	-1.39 (0.68)*	0.09
	L.Ins-ACC	1.07 (0.67)	
	PCC-ACC	-0.90 (0.71)	
Inhibitory Control (Parent Report)	L.Ins-L.Put	-0.73 (0.30)*	0.24
	vmPFC-R.OFC	-0.54 (0.30)	
	L.Caud-L.NAcc	0.69 (0.29)*	
	L.Amyg-R.Amyg	0.67 (0.30)*	
	R.Caud-L.Caud	-0.71 (0.30)*	
	ACC-PCC	-0.93 (0.30)**	
	vmPFC-ACC	-0.54 (0.30)	
Depression (Child Report)	L.Amyg-R.Amyg	-0.23 (0.09)*	0.06
Depression (Parent Report)	-	-	0
Familial Risk for Substance Use Problems	vmPFC-R.OFC	0.09 (0.04)*	0.24
	L.Ins-L.OFC	-0.09 (0.04)*	
	R.Ins-L.Ins	-0.08 (0.04)	
	R.Put-R.Caud	-0.09 (0.04)*	
	R.Caud-L.Caud	-0.06 (0.04)	
	R.Caud-R.NAcc	-0.11 (0.04)**	
	R.Caud-L.Ins	-0.11 (0.04)*	
	R.Ins-L.OFC	0.05 (0.04)	
	L.Ins-R.Put	0.05 (0.04)	
	L.Ins-L.Amyg	0.07 (0.04)	
	L.Ins-ACC	-0.04 (0.04)	
	ACC-PCC	-0.08 (0.04)	
	PCC-ACC	0.03 (0.04)	
Follow-Up Alcohol Expectancies	R.OFC-L.OFC	2.44 (1.45)	0.27
	R.Put-L.Ins	1.73 (1.59)	
	L.Amyg-L.NAcc	-4.27 (1.67)*	
	R.Ins-R.Amyg	2.87 (1.54)	
	ACC-PCC	-4.24 (1.71)*	

* p < .05, ** p < .01, *** p < .001

S13. Descriptive Statistics of Behavioral Measures

Measure	Included Participants (M (SD))	Excluded Participants (M/SD or %)
Age	11.35 (1.46)	10.57 (1.35)
Sex	57% Female	58% Female
Delay Discounting Log(K)	-4.65 (2.72)	-4.78 (3.82)
BAS Reward Responsivity (Child Report)	16.96 (2.50)	17.06 (2.96)
BAS Reward Drive (Child Report)	10.31 (2.74)	10.59 (2.75)
BAS Fun Seeking (Child Report)	12.16 (2.30)	12.35 (2.57)
Pleasure Scale for Children	47.93 (15.87)	50.62 (17.20)
EATQ Pleasure Sensitivity	14.39 (5.97)	14.94 (6.53)
BAS Reward Responsivity (Parent Report)	17.58 (2.15)	17.75 (2.86)
BAS Reward Drive (Parent Report)	11.53 (2.68)	11.26 (2.76)
BAS Fun Seeking (Parent Report)	11.83 (2.65)	12.08 (2.77)
EATQ Inhibitory Control (Child Report)	26.50 (5.76)	26.01 (6.04)
EATQ Inhibitory Control (Parent Report)	13.58 (3.22)	13.22 (3.52)
CDI (Child Report)	6.68 (7.03)	7.03 (7.74)
MFQ (Child Report)	7.70 (10.44)	7.70 (10.16)
CDI (Parent Report)	4.12 (5.11)	4.70 (4.72)
MFQ (Parent Report)	4.55 (6.59)	3.68 (5.35)
Familial Risk of Substance Use Disorder	25% Maternal SUD History Rate	22% Maternal SUD History Rate

References

Abraham, Alexandre, Fabian Pedregosa, Michael Eickenberg, Philippe Gervais, Andreas Mueller, Jean Kossaifi, Alexandre Gramfort, Bertrand Thirion, and Gael Varoquaux. 2014. "Machine Learning for Neuroimaging with Scikit-Learn." Frontiers in Neuroinformatics 8. https://doi.org/10.3389/fninf.2014.00014.

Avants, B.B., C.L. Epstein, M. Grossman, and J.C. Gee. 2008. "Symmetric Diffeomorphic Image Registration with Cross-Correlation: Evaluating Automated Labeling of Elderly and Neurodegenerative Brain." Medical Image Analysis 12 (1): 26–41. https://doi.org/10.1016/j.media.2007.06.004.

Behzadi, Yashar, Khaled Restom, Joy Liau, and Thomas T. Liu. 2007. "A Component Based Noise Correction Method (CompCor) for BOLD and Perfusion Based fMRI." NeuroImage 37 (1): 90–101. https://doi.org/10.1016/j.neuroimage.2007.04.042.Cox, Robert W., and James S. Hyde. 1997. "Software Tools for Analysis and Visualization of fMRI Data." NMR in Biomedicine 10 (4-5): 171–78. https://doi.org/10.1002/(SICI)1099-1492(199706/08)10:4/5<171::AID-NBM453>3.0.CO;2-L.

Costa Dias, T. G., Iyer, S. P., Carpenter, S. D., Cary, R. P., Wilson, V. B., Mitchell, S. H., Nigg, J. T., & Fair, D. A. (2015). Characterizing heterogeneity in children with and without ADHD based on reward system connectivity. *Developmental Cognitive Neuroscience*, *11*, 155–174. https://doi.org/10.1016/j.dcn.2014.12.005

Drysdale, A. T., Grosenick, L., Downar, J., Dunlop, K., Mansouri, F., Meng, Y., Fetcho, R. N., Zebley, B., Oathes, D. J., Etkin, A., Schatzberg, A. F., Sudheimer, K., Keller, J., Mayberg, H. S., Gunning, F. M., Alexopoulos, G. S., Fox, M. D., Pascual-Leone, A., Voss, H. U., … Liston, C. (2017). Resting-state connectivity biomarkers define neurophysiological subtypes of depression. *Nature Medicine*, *23*(1), 28–38. https://doi.org/10.1038/nm.4246

Esteban, Oscar, Ross Blair, Christopher J. Markiewicz, Shoshana L. Berleant, Craig Moodie, Feilong Ma, Ayse Ilkay Isik, et al. 2018. "FMRIPrep." Software. Zenodo. https://doi.org/10.5281/zenodo.852659.

Esteban, Oscar, Christopher Markiewicz, Ross W Blair, Craig Moodie, Ayse Ilkay Isik, Asier Erramuzpe Aliaga, James Kent, et al. 2018. "fMRIPrep: A Robust Preprocessing Pipeline for Functional MRI." Nature Methods. https://doi.org/10.1038/s41592-018-0235-4.

Fonov, VS, AC Evans, RC McKinstry, CR Almli, and DL Collins. 2009. "Unbiased Nonlinear Average Age-Appropriate Brain Templates from Birth to Adulthood." NeuroImage 47, Supplement 1: S102. https://doi.org/10.1016/S1053-8119(09)70884-5.

Gorgolewski, K., C. D. Burns, C. Madison, D. Clark, Y. O. Halchenko, M. L. Waskom, and S. Ghosh. 2011. "Nipype: A Flexible, Lightweight and Extensible Neuroimaging Data Processing Framework in Python." Frontiers in Neuroinformatics 5: 13. https://doi.org/10.3389/fninf.2011.00013.

Gorgolewski, Krzysztof J., Oscar Esteban, Christopher J. Markiewicz, Erik Ziegler, David Gage Ellis, Michael Philipp Notter, Dorota Jarecka, et al. 2018. "Nipype." Software. Zenodo. https://doi.org/10.5281/zenodo.596855.

Greve, Douglas N, and Bruce Fischl. 2009. "Accurate and Robust Brain Image Alignment Using Boundary-Based Registration." NeuroImage 48 (1): 63–72. https://doi.org/10.1016/j.neuroimage.2009.06.060.

Huntenburg, Julia M. 2014. "Evaluating Nonlinear Coregistration of BOLD EPI and T1w Images." Master's Thesis, Berlin: Freie Universität. http://hdl.handle.net/11858/00-001M-0000-002B-1CB5-A.

Jenkinson, Mark, Peter Bannister, Michael Brady, and Stephen Smith. 2002. “Improved Optimization for the Robust and Accurate Linear Registration and Motion Correction of Brain Images.” NeuroImage 17 (2): 825–41. https://doi.org/10.1006/nimg.2002.1132.

Jenkinson, Mark, and Stephen Smith. 2001. “A Global Optimisation Method for Robust Affine Registration of Brain Images.” Medical Image Analysis 5 (2): 143–56. https://doi.org/10.1016/S1361-8415(01)00036-6.

Kashyap, R., Bhattacharjee, S., Yeo, B. T. T., & Chen, S. H. A. (2020). Maximizing dissimilarity in resting state detects heterogeneous subtypes in healthy population associated with high substance use and problems in antisocial personality. *Human Brain Mapping*, *41*(5), 1261–1273. https://doi.org/10.1002/hbm.24873

Lanczos, C. 1964. “Evaluation of Noisy Data.” Journal of the Society for Industrial and Applied Mathematics Series B Numerical Analysis 1 (1): 76–85. https://doi.org/10.1137/0701007.

Liang, S., Deng, W., Li, X., Greenshaw, A. J., Wang, Q., Li, M., Ma, X., Bai, T.-J., Bo, Q.-J., Cao, J., Chen, G.-M., Chen, W., Cheng, C., Cheng, Y.-Q., Cui, X.-L., Duan, J., Fang, Y.-R., Gong, Q.-Y., Guo, W.-B., … Li, T. (2020). Biotypes of major depressive disorder: Neuroimaging evidence from resting-state default mode network patterns. *NeuroImage: Clinical*, *28*, 102514. https://doi.org/10.1016/j.nicl.2020.102514

Power, Jonathan D., Anish Mitra, Timothy O. Laumann, Abraham Z. Snyder, Bradley L. Schlaggar, and Steven E. Petersen. 2014. “Methods to Detect, Characterize, and Remove Motion Artifact in Resting State fMRI.” NeuroImage 84 (Supplement C): 320–41. https://doi.org/10.1016/j.neuroimage.2013.08.048.

Satterthwaite, Theodore D., Mark A. Elliott, Raphael T. Gerraty, Kosha Ruparel, James Loughead, Monica E. Calkins, Simon B. Eickhoff, et al. 2013. “An improved framework for confound regression and filtering for control of motion artifact in the preprocessing of resting-state functional connectivity data.” NeuroImage 64 (1): 240–56. https://doi.org/10.1016/j.neuroimage.2012.08.052.

Treiber, Jeffrey Mark, Nathan S. White, Tyler Christian Steed, Hauke Bartsch, Dominic Holland, Nikdokht Farid, Carrie R. McDonald, Bob S. Carter, Anders Martin Dale, and Clark C. Chen. 2016. “Characterization and Correction of Geometric Distortions in 814 Diffusion Weighted Images.” PLOS ONE 11 (3): e0152472. https://doi.org/10.1371/journal.pone.0152472.

Tustison, N. J., B. B. Avants, P. A. Cook, Y. Zheng, A. Egan, P. A. Yushkevich, and J. C. Gee. 2010. “N4ITK: Improved N3 Bias Correction.” IEEE Transactions on Medical Imaging 29 (6): 1310–20. https://doi.org/10.1109/TMI.2010.2046908.

Wang, Sijia, Daniel J. Peterson, J. C. Gatenby, Wenbin Li, Thomas J. Grabowski, and Tara M. Madhyastha. 2017. “Evaluation of Field Map and Nonlinear Registration Methods for Correction of Susceptibility Artifacts in Diffusion MRI.” Frontiers in Neuroinformatics 11. https://doi.org/10.3389/fninf.2017.00017.

Zhang, Y., M. Brady, and S. Smith. 2001. “Segmentation of Brain MR Images Through a Hidden Markov Random Field Model and the Expectation-Maximization Algorithm.” IEEE Transactions on Medical Imaging 20 (1): 45–57. https://doi.org/10.1109/42.906424.

Zhu, T., Becquey, C., Chen, Y., Lejuez, C. W., Li, C.-S. R., & Bi, J. (2022). Identifying alcohol misuse biotypes from neural connectivity markers and concurrent genetic associations. *Translational Psychiatry*, *12*(1), 1–9. https://doi.org/10.1038/s41398-022-01983-1
